# A systematic review and meta-analysis on early-childhood-caries global data

**DOI:** 10.1186/s12903-024-04605-y

**Published:** 2024-07-24

**Authors:** Anastasia Maklennan, R. Borg-Bartolo, R. J. Wierichs, M. Esteves-Oliveira, G. Campus

**Affiliations:** 1 Department of Restorative, Preventive and Pediatric Dentistry, School of Dental Medicine, University of Bern, Freiburgstrasse 7, Bern, 3010 Switzerland; 2 Department of Conservative Dentistry, Periodontology and Endodontology, Oral Medicine and Maxillofacial Surgery, University Centre of Dentistry, University Hospital Tübingen, Tübingen, Germany; 3 Department of Surgery, Microsurgery and Medicine Sciences, School of Dentistry, University of Sassari, Viale San Pietro 3/c, Sassari, 07100 Italy; 4 Graduate School for Health Sciences, University of Bern, Bern, Switzerland

**Keywords:** Epidemiology, Caries, Community dentistry, Dental public health, Child dentistry, Meta-analysis

## Abstract

**Objectives:**

The present study systematically reviewed and provided a meta-analysis on early childhood caries (ECC) global prevalence and its association with socioeconomic indicators, both geographical and regarding unemployment rate, national income as well as income inequalities.

**Methods:**

Only cross-sectional or cohort studies covering ECC prevalence and experience in children younger than 71 months, reporting sample size, diagnostic criteria and conducted in urban and rural communities were considered. No language restriction was selected. Studies published from 2011 to 2022 available in PubMed, Web of Science, Embase and Open Grey literature were retrieved by ad hoc prepared search strings. The meta-analyses were conducted for both overall ECC prevalence and experience stratified by country of publication as well as measures of socioeconomic indicators using a random effects model using STATA 18^®^.

**Results:**

One hundred publications reporting ECC data from 49 countries (published from 2011 to 2022) were included and summarized by meta-analysis. The lowest prevalence was reported in Japan (20.6%) and Greece (19.3%). The global estimated random-effect pooled prevalence of ECC was 49% (95%CI: 0.44–0.55). The random-effect pooled caries prevalence (ECC) was 34% (95%CI: 02.20–0.48) (Central/South America), 36% (95%CI: 0.25–0.47) (Europe), 42% (95%CI: 0.32–0.53) (Africa), 52% (95%CI: 0.45–0.60) (Asia-Oceania), 57% (95%CI: 0.36–0.77) (North America) and 72% (95%CI: 0.58–0.85) (Middle East). When stratified by gross national income (GNI) the ECC prevalence ranged from 30% ($20,000-$39,999) to 57% in countries with the lowest GNI (<$5000). Stratification by inequality index (Gini index) resulted in an ECC prevalence range of 39% (low inequality) to 62% (no inequality), while for life expectancy the ECC prevalence ranged from 28% in countries with the highest life expectancy (< 80 years) to 62% in countries with 71–75 years life expectancy.

**Discussion:**

Within the limitations of this study (lack of certainty about the results as many countries are not represented and lack of uniformity in prevalence and experience data represented), results from 49 different countries reported a wide range of ECC prevalence. These reports indicated persisting high worldwide distribution of the disease. Both ECC prevalence and experience were associated with geographical areas and GNI.

**Registration:**

PROSPERO: CRD-42,022,290,418.

**Supplementary Information:**

The online version contains supplementary material available at 10.1186/s12903-024-04605-y.

## Introduction

ECC (Early Childhood Caries) is defined as the presence of one or more decayed (non-cavitated or cavitated lesions), missing (due to caries), or filled tooth surfaces in any primary tooth in a child under the age of six [1]. Although preventable, ECC remains a global health problem leading to negative impacts on a child’s development and growth patterns as well as causing oral health–related quality of life issues, such as inadequate nutrition. Despite recent efforts in lowering caries prevalence through various caries prevention programs, the overall trend is still high [2, 3]. Recent studies show that up to half of the toddler population worldwide is still being affected by this chronic disease [4]. As for general caries disease a multifactorial etiopathogenetic development model has been described for ECC [5, 6] with socioeconomic disparities playing a major role [7]. Prevalence in countries that are often even in the same continent, fluctuate hugely mostly due to economic conditions, distribution of wealth, and even access to basic human needs, such as childhood education [8]. These differences are reflected in human general as well as in oral health [9–12]. However, there have been only few studies looking at the figures of Early Childhood Caries in countries with different levels of economic and human development status [2, 3]. Moreover, only fragmentary knowledge exists about the correlation to factors such as wealth inequality within a nation index (Gini coefficient) and/or gross national income per capita/year (GNI) of several countries. The numbers of ECC do not fall as rapidly as anticipated especially among socioeconomically deprived children [13], therefore, it is critical to investigate the association between background social and economic factors and ECC prevalence/experience worldwide. Starting from these premises, this systematic review and meta-analysis were aimed to synthesize existing research findings regarding ECC prevalence and experience globally over the last ten years and to describe its distribution by country as well as its links to various socio-economic indicators. The outcomes may help to generate a better understanding of ECC and disparities at a global level, which may then facilitate more targeted and culturally safe approaches to assist policy stakeholders in implementing preventive programs against ECC.

## Materials and methods

This systematic review was conducted following the preferred reporting items for systematic reviews and meta-analysis (PRISMA2020) guidelines and the protocol was registered at the international prospective register of systematic reviews PROSPERO, registration: CRD-42,022,290,418).

### Eligibility criteria

Only cross-sectional or cohort studies covering ECC prevalence and experience in children younger than 71 months, reporting sample size, diagnostic criteria and conducted in urban and rural communities were considered. Studies published from 2011 to September 2022 were considered. Studies regarding children with congenital anomalies, dentofacial anomalies and medically compromised children were discarded. Primary outcomes of the included studies were the prevalence and experience of ECC. No language restriction was selected, as the authors are fluent in English, French, Spanish, Italian, Portuguese, German and Russian. For all other languages an artificial-intelligence-based tool named DeepL Translate was used.

### Data sources and strategy

Detailed search strategies and search strings were appropriately created using Boolean terms. Electronic databases PubMed, Embase, Scopus and Open Grey literature (http://www.opengrey.eu) were searched (10.10.2022). Additional hand search was performed in case no prevalence data was found after the systematic search for a specific country (Supplementary material. Appendix Table 1).

### Study selection

Three authors (AM, MEO, GC) independently reviewed titles and abstracts, excluding those not meeting the inclusion criteria. The reviewers were not blinded to the identity of the journal names, article authors, institutions, or the results of the research. The full texts of the selected papers were assessed and agreement concerning study inclusion was made by discussion between the three authors.

### Data extraction

Two authors performed data extraction independently and in duplicate top identify areas of discrepancy, resolve those and assess inter-rater agreement (AM, MEO). Inter-rater agreement (kappa = 0,867, which equated to ‘Almost perfect agreement’).

The following data was collected in pilot-tested excel files: author/title/year of study, study affiliation, study type and setting, design of the study, number/age/gender of patients as well as caries prevalence (percentage) and experience (dmft index, if “m” was not considered in the publication only dft was used).

### Quality assessment

A customized quality assessment tool developed by The National Heart, Lung and Blood Institute for Observational Cohort and Cross-sectional studies, Case-Control studies and Controlled-Intervention studies (https://www.nhlbi.nih.gov/health-topics/study-quality-assessment-tools) was used. Quality of the papers was assessed according to the following criteria: 0–6 poor quality, 7–10 fair quality, 11–14 high quality. The assessment was carried out by one trained author (AM).

### Data synthesis

Studies with a cross-sectional design, including the final sample size, the proportion of individuals who had ECC and/or the mean number of teeth affected by caries, were included in the meta-analysis. If prevalence or means for two or more age-subgroups were reported in the same paper, the overall prevalence or mean dmft was used. The primary outcomes were caries prevalence (%, dmft > 0) and experience (mean dmft) and their association to socioeconomical conditions of the countries: geographical area, GNI (Gross National Income *per* capita/year), Gini (wealth inequality within a nation index) coefficient, unemployment rate and life expectancy. Countries were categorized into six world regions; Africa, North America, Central/South America, Asia-Oceania, Europe and the Middle East. The GNI, Gini coefficient, unemployment rate and life expectancy were retrieved from the World Bank website (https://data.worldbank.org*).* Gini index of 0 represents perfect equality, while an index of 100 implies perfect inequality. GNI was categorized by authors base on existing categorization (https://blogs.worldbank.org/opendata/new-world-bank-country-classifications-income-level-2020-2021*)* as follows: countries with the income of < 5000 USD *per capita*/year; >5000-<10,000 USD *per capita*/year; >10,000-<20,000 USD; >20,000-<40,000 USD and > 40,000 USD. The Gini coefficient was categorized by authors based existing on the categorization for Gini-index [14] as follow: <32 no inequalities, 32.1–35 low inequalities, 35.1–40 medium inequalities and > 40 high inequalities. Gini index data for Hong Kong, Qatar and Cambodia was not available. They were retained as one group in the meta-analyses stratified by Gini index. Unemployment Rate was categorized as 0.1-<3 low; 3.1-<6.0 medium; 6.1-<10 high and > 10 very high (https://data.worldbank.org). Solely for a qualitative analysis also life expectancy data (in years) was collected and categorized according to existing categorization (https://ourworldindata.org/life-expectancy*)* as 53–70 yy, 71–75 yy, 76–80 yy and > 80 yy. ECC prevalence was categorized in four groups as percentage based on the existing categorization [15, 16] as follows ≤ 20% - low prevalence, 21–40% - medium prevalence, 41–60% - medium-high prevalence and > 60% - high prevalence [17]. To create a graphic overview of the worldwide ECC prevalence in each country was summarized including only the most recent study (most recent prevalence value) from each country (*n* = 49), categorized according to different ranges and a respective color scheme in world map. The dmft index of ECC was categorized by authors and literature [16] as follows < 1 (low experience), 1.01-2 (medium experience), 2.01-4 (medium-high experience) and > 4 (high experience) considering the distribution of caries experience reported in the include papers. Data were presented as a proportion for caries prevalence as 95%CI and for caries experience as means ± SD. The association between caries prevalence and experience and socio-economic indicators was evaluated by contingency tasks. Heterogeneity was estimated using the I^2^ statistic, a statistic which describes the percentage of variation between studies due to heterogeneity and not to chance, and a 95% prediction interval. Meta-analysis was performed using the random-effects method with 95% confidence interval to take into consideration existing heterogeneity between studies. Funnel plots or other tests to evaluate publication bias are not being presented in the current review. Publication bias assumes that studies with positive results are more frequently published than studies with negative results. However, this assumption does not necessarily hold for studies of prevalence [35]. StataSE18^®^ was used for the statistical analysis.

## Results

### Studies selection and characteristics of the included studies

**Fig. 1:**
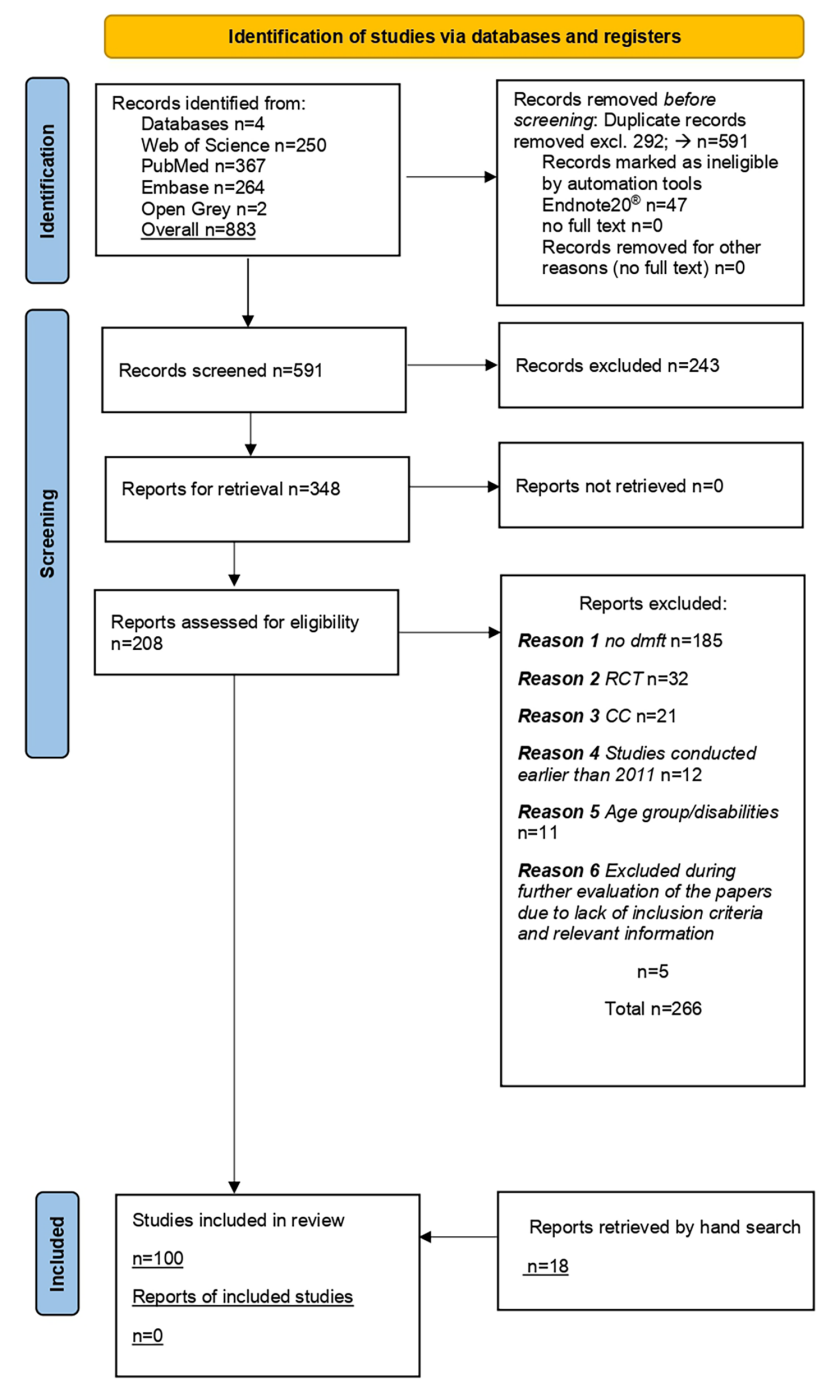
PRISMA 2020 Flowchart of the search strategy and identification process of the papers. Legend: dmft - decayed, missing, filled teeth; RCT - randomized clinical trial; CC - cross sectional studies

Overall, 881 papers were retrieved, 292 duplicates were excluded and 589 were selected. After titles and abstracts evaluation 348 reports assessed for eligibility, 266 were excluded (Supplementary material. Appendix Table 2). Remaining 82 were obtained in full text format, as well as additional 18 papers were retrieved by hand search (Fig. 1).

According to the year of publication, 13 studies were published between 2011 and 2013, 31 studies from 2014 to 2016, 26 from 2017 to 2019 and 30 from 2020 to 2022. One publication reported data on early childhood caries prevalence from more than one country [18], thus, data was entered separately for each country reported in the study.

### Quality assessment

Most studies (*n* = 68) were classified as being of fair quality, while three papers and twenty-nine studies were ranked as being of high and poor quality, respectively. Quality assessment relied on sample size justification or power analysis and lacking those criteria affected the quality outcome of the papers. However, in almost all publications, research questions, study populations and outcome measures were clearly defined (Supplementary Material. Appendix Table 3).

### Synthesis of results

**Fig. 2:**
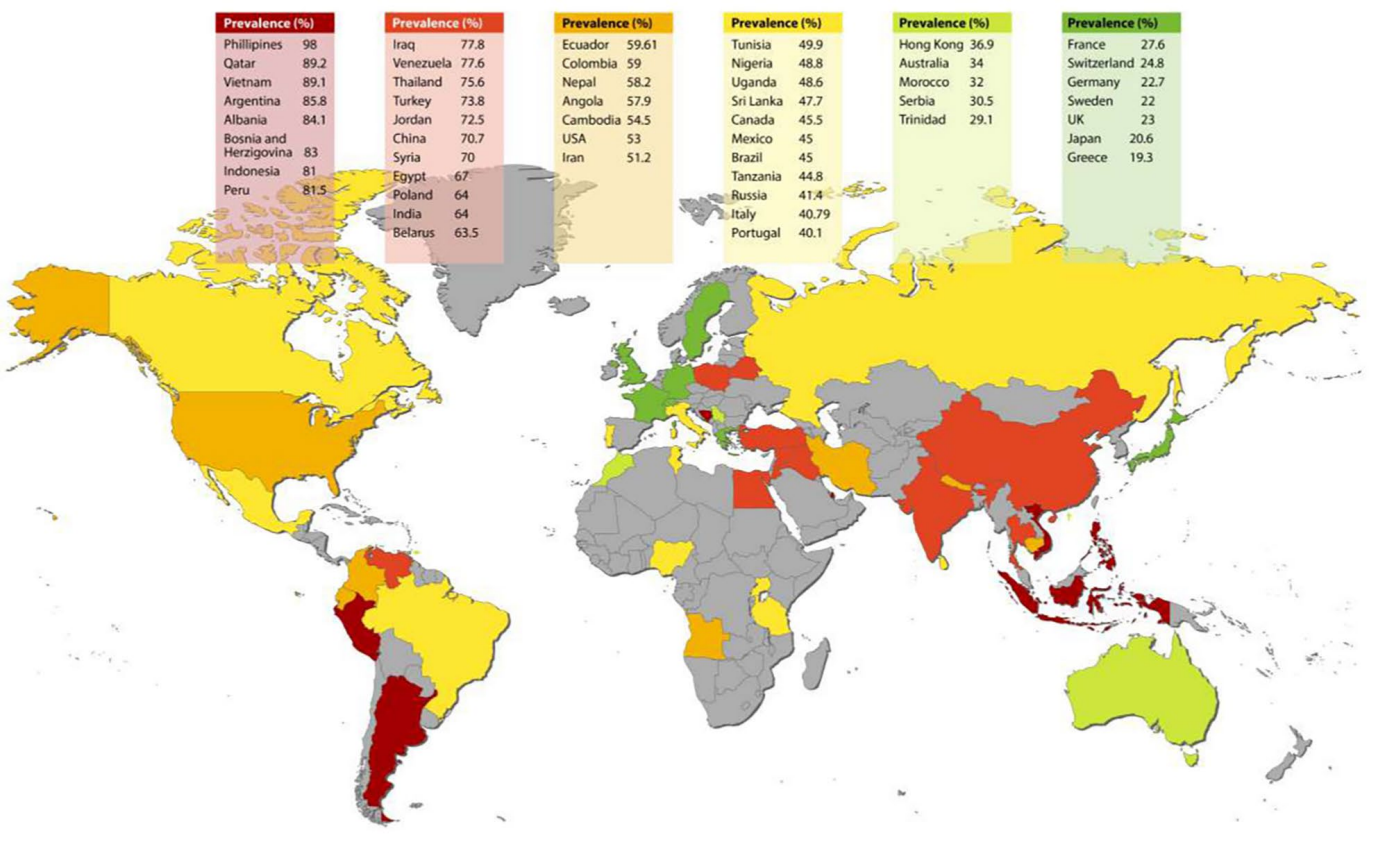
Worldwide ECC prevalence map. Prevalence was color coded as following: (**a**) < 29% - low prevalence (dark green), (**b**) 29.1–39% - medium low prevalence (light green), (**c**) 40-49.9% - medium prevalence (yellow), (**d**) 50–60% - medium high prevalence (orange), 60–80% - high prevalence (light red), (**e**) > 80% - very high prevalence (dark red)

From the included papers (*n* = 100), ECC prevalence was reported in 67 of them. One paper reported data from 3 different countries [18] (Supplementary material. Appendix Table 4). Worldwide highest ECC prevalence was reported for the Philippines (98.0%) [18], while the lowest were in Japan (20.6%) [19] and Greece (19.3%) [18]. The highest ECC prevalence for North America was reported in the USA (53.0%) [20], for South America in Argentina (85.8%) [21], for Europe in Albania (84.1%) [22] and for Africa in Angola (57.9%) [23] (Fig. 2).

**Fig. 3:**
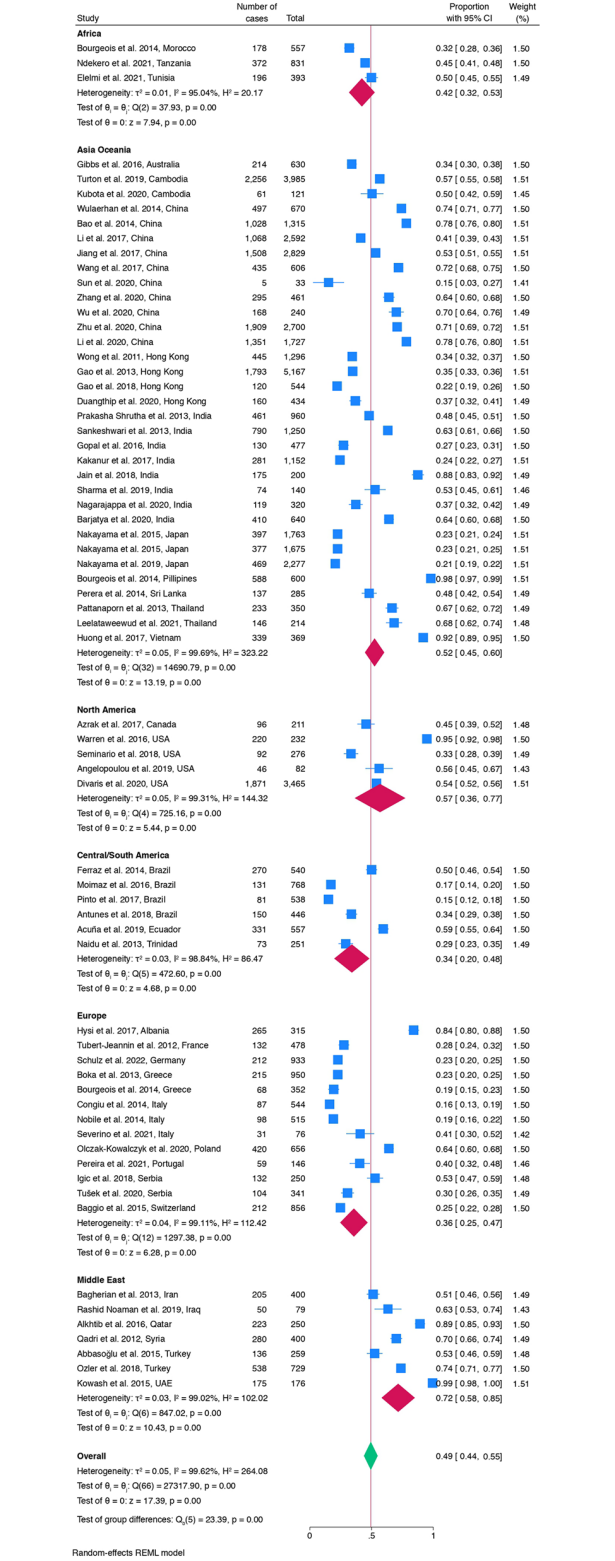
Forest plot of the pooled ECC prevalence stratified by geographical area

Meta-analysis of ECC prevalence reported high heterogeneity between the studies (I^2^ = 99%, 95% prediction interval 0.03–0.96). The global estimated random-effect pooled prevalence of ECC was 49% (95%CI: 0.44–0.55). The random-effect pooled caries prevalence (ECC) was 34% (95%CI: 02.20–0.48) (Central/South America), 36% (95%CI: 0.25–0.47) (Europe), 42% (95%CI: 0.32–0.53) (Africa), 52% (95%CI: 0.45–0.60) (Asia-Oceania), 57% (95%CI: 0.36–0.77). (North America) and 72% (95%CI: 0.58–0.85) (Middle East) (Fig. 3).

Similarly, to the ECC prevalence, the highest dmft was also reported in the Philippines [18] (mean 12.03 ± 5.2) and the lowest in Japan [19] (0.1 ± 0.7). The highest dmft for North America was reported in the USA (8.0 ± NR) [20], for South America in Colombia (mean 5.5 ± 9.0) [24] for Europe, in Bosnia and Herzegovina (mean 6.79 ± 5.25) [25] and for Africa in Morocco (mean 5.01 ± 5.5) [18].

**Fig. 4:**
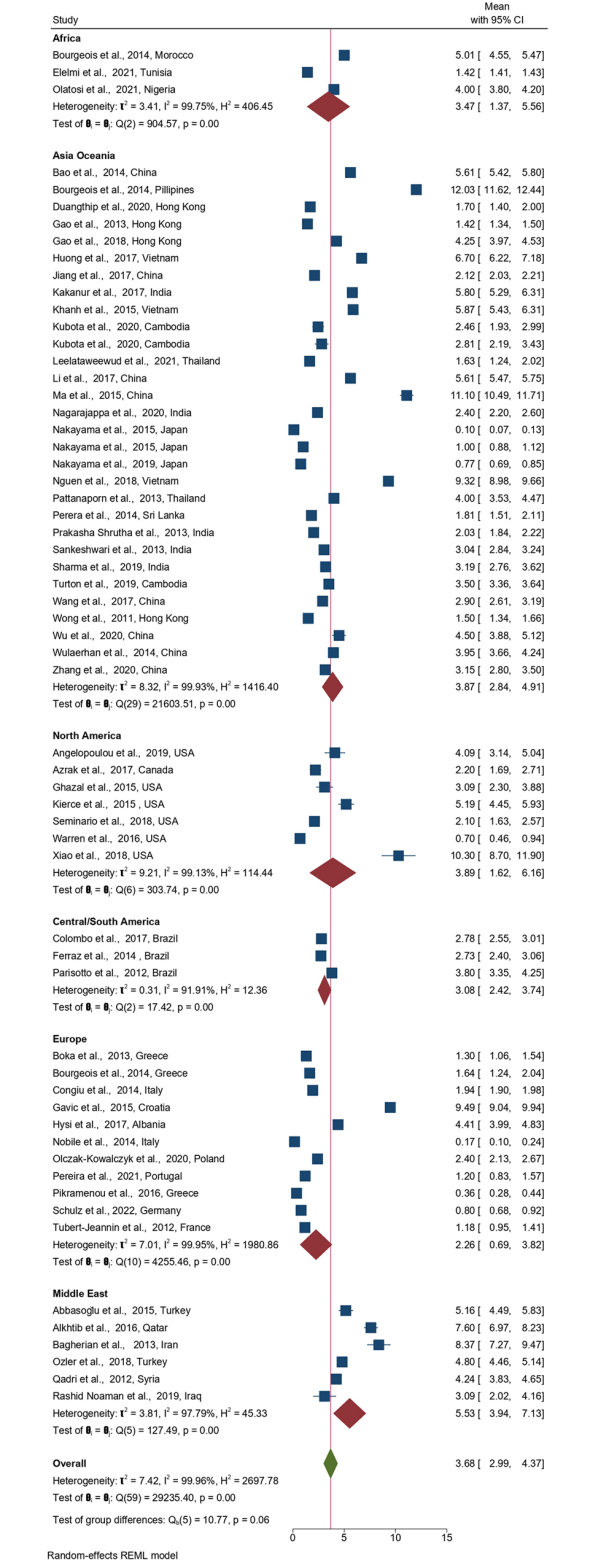
Forest plot of the pooled ECC experience stratified by geographical area

Meta-analysis of mean dmft reported high heterogeneity (I^2^ = 99%, 95% prediction interval − 0.90–8.27). The global estimated random-effect pooled mean dmft was 3.68 teeth (95%CI: 2.99–4.37); 2.26 teeth (95%CI: 0.69–3.82) (Europe), 3.08 teeth (95%CI: 2.42–3.74 (Central/South America), 3.47 teeth (95%CI: 1.37–5.56) (Africa), 3.87 teeth (95%CI: 2.84–4.91) (Asia-Oceania), 3.89 teeth (95%CI: 1.62–6.16) (North America), 5.53 teeth (95%CI: 3.94–7.13) (Middle East) (Fig. 4).

Bivariate analysis of the pooled data about the association of early childhood caries prevalence/experience with socioeconomic indicators is presented in the Supplementary file (Supplementary material. Appendix Tables 6a, b,c, d,e/Tables 7a, b,c, c,e). High ECC prevalence was statistically significantly associated with geographical areas (Supplementary material. Appendix Table 6a, *p* = 0.02) being the highest in Asia, with low GNI was (Supplementary material. Appendix Table 6b, *p* < 0.01) and with higher unemployment rate (Supplementary Material. Appendix Table 6d, *p* = 0.03).

**Fig. 5:**
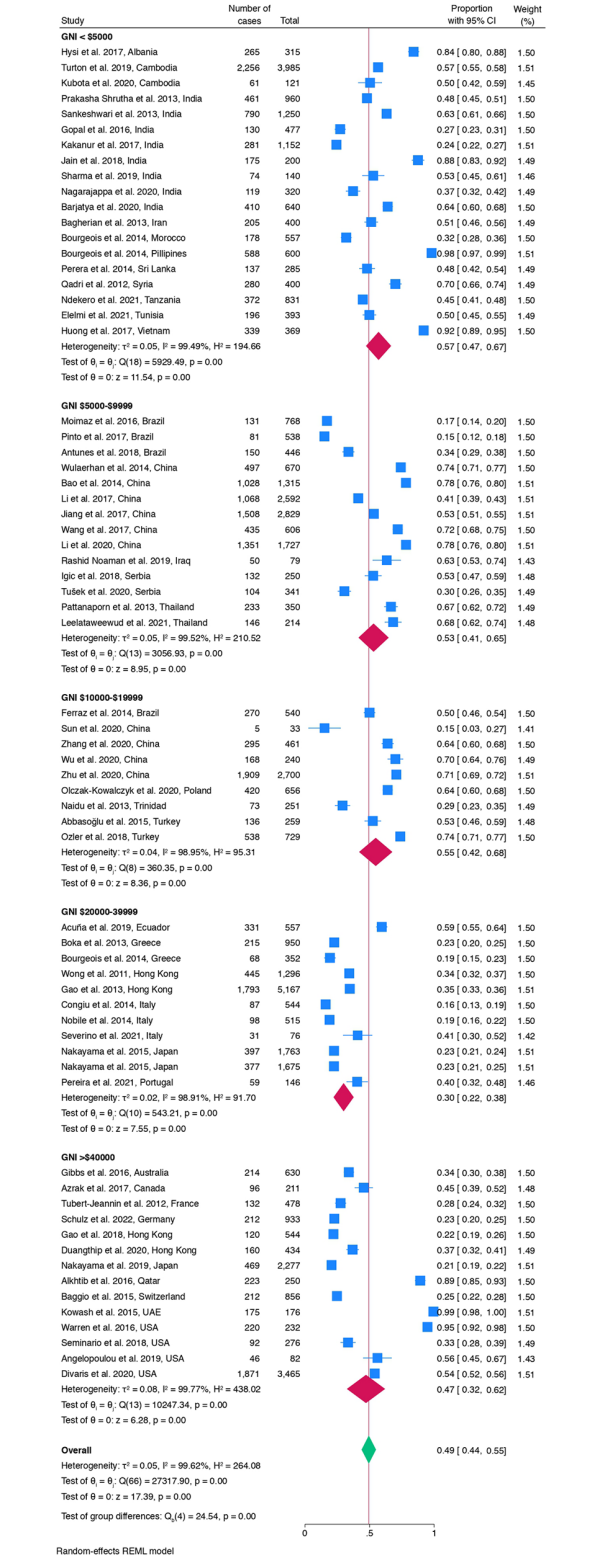
Forest plot of the pooled ECC prevalence stratified by GNI

When stratified by GNI the ECC prevalence ranged from 30% ($20,000-$39,999) to 57% in countries with the lowest GNI (<$5000) (Fig. 5).

Stratification by Gini index resulted in an ECC prevalence range of 39% (low inequality) to 62% (no inequality), while for life expectancy the ECC prevalence ranged from 28% in countries with the highest life expectancy (< 80 years) to 62% in countries with 71-75-year- life expectancy.

High dmft means were statistically significantly associated with geographical areas (Supplementary material. Table 7a, *p* < 0.01), being the highest in Asia, with low GNI (Table 7b, *p* = 0.04). Unemployment rate was not statistically (Supplementary Material. Table 7d, *p* = 0.42) associated with dmft, even if a tendency in high dmft means in countries with higher unemployment rates were reported.

**Fig. 6:**
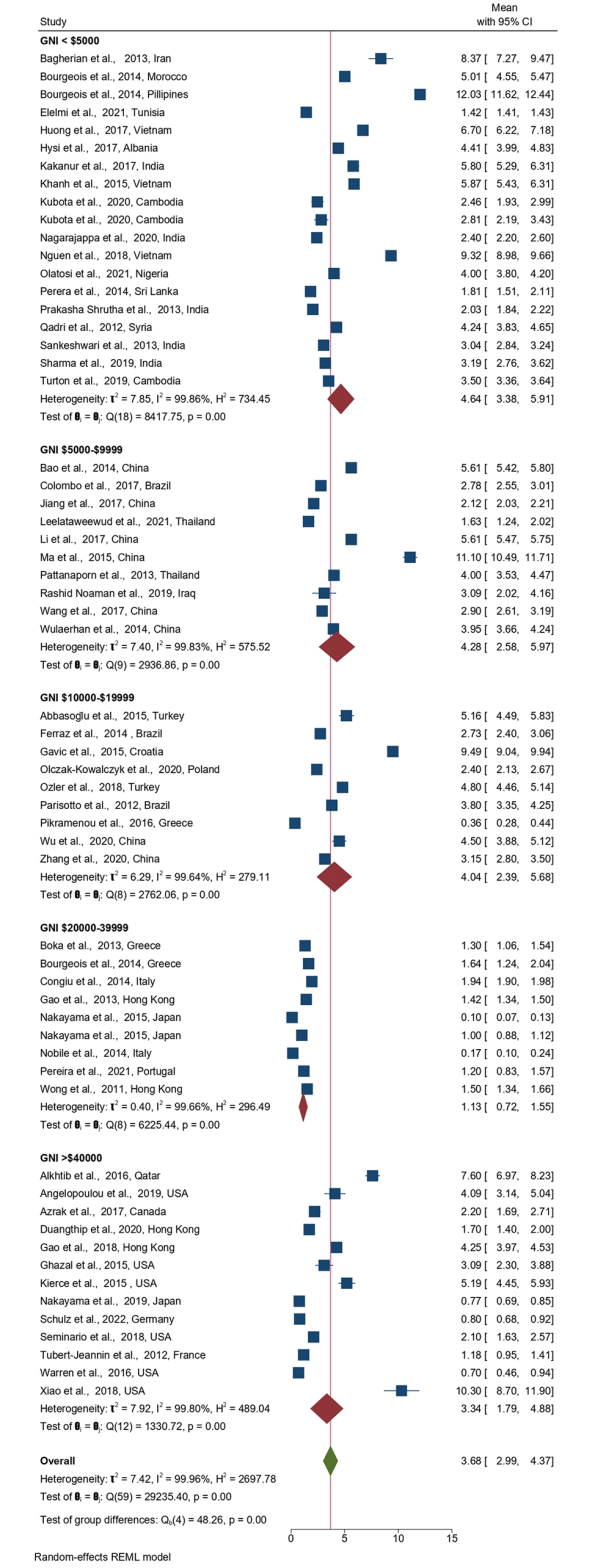
Forest plot of the pooled ECC experience stratified by GNI

The lowest pooled mean dmft was reported for countries with GNI $20,000-$39,999 while countries with GNI < $5000 reported the highest pooled mean dmft = 4.64 teeth (Fig. 6).

Countries with the highest life expectancy (> 80 years) presented the lowest pooled mean dmft = 1.34 teeth while the highest dmft (5.18 teeth) was found in countries with a life expectancy between 71 and 75 years. With regards to Gini index, the lowest mean dmft (1.62 teeth) was reported for countries with the low inequality while the highest mean dmft was reported for countries with no inequalities (dmft = 3.95 teeth). Meta-regression and subgroup analysis of regions of the world and year of publication did not explain heterogeneity.

## Discussion

The aim of this systematic review was to identify and analyse the worldwide data on ECC prevalence and experience and its association with socioeconomic factors worldwide. ECC has significant influence on individuals, families and societies. The disease affects primary teeth and permanent teeth and influences general health and quality of life across the entire life course. ECC links with other frequent diseases of childhood, primarily due to risk factors shared with other noncommunicable diseases (NCDs) such as high sugar intake, and the disease relates to other health conditions such as obesity [26]. Dental caries can lead to abscesses and cause toothache, which may compromise ability to eat and sleep and restrict life activity of children. Severe dental caries is associated with poor growth [26, 27].

Moreover, ECC is an economic burden to the family and society; treatment of ECC under general anaesthesia for extensive dental repair is especially costly. *However*,* as primary teeth are exfoliated due to the child’s growth*,* ECC has previously not been considered importan*t [26]. As most of the epidemiological studies on caries prevalence have been carried out on 12-year-old children, there is still scarce information in the literature on effectiveness of the preventive measures in reducing caries also in young children under 5 years of age. As the treatment of severe ECC often involves complex treatment measures (i.e. general anaesthesia) and generate a great psychological and financial impact on supporting state health systems, on public health systems, it is important to summarise the currently available data on the prevalence of caries worldwide to assist policy makers [28]. The study revealed that early childhood caries affects as much as 49% of preschool population (95%CI: 0.44–0.55). The distribution of ECC is global and varies according to the geographical area. The present review reported the prevalence of ECC to be lower than the global pooled prevalence in Africa, while in Asia/Oceania and Europe it was higher than world estimate. For both caries prevalence and experience the highest figures were reported in countries with the lowest GNI. An interesting finding is that a large variation of ECC prevalence was reported within some countries such as Thailand, Brazil, Japan [19, 29–31]. These differences indicate high variation in methods and area of data collection. In a recent systematic review and meta-analysis [4] the main source of heterogeneity was reported between countries. However, differences in caries burden can also be observed within one country as for example in the USA. A very high ECC prevalence of 80% was reported [32], however, it was assessed only in a group of indigenous Indian American children, which is not representative for such a large country. A recent study [3] pointed out similar problems in substantial caries burden inequality across countries in sub-Saharan Africa, in toddler population aged 1 to 4 yy. According to the present review caries prevalence remained unchanged in the last 10 years, except for Brazil, where significant changes in caries prevalence was observed showing a downward trend over the 10-year period [33–35]. These examples indicate that there is a strong link to socioeconomic indicators within each country.

Those differences between countries indicate that there are certain factors affecting the oral health of children younger than 3 years of age.

This is the first registered systematic review with a meta-analysis of the pooled prevalence and experience of ECC assessing data available in the last 10 years and its association with various socio-economic factors.

An association between several socioeconomic indicators (low education, parental occupational background, income) and high caries experience has been shown in the study by Schwendicke et al. [2]. However, up to now the association of high caries experience with other variables like (unemployment rate, life expectancy) and especially the national income and income inequalities indexes (GNI/Gini), as defined by the World Bank has not been conducted. Apparently, inequalities of income distribution, is unexpectedly not really impacting caries experience, but Gross Nation Income (GNI) does. Meaning in wealthier countries, independently of how the income is distributed, the general abundance of resources might positively influence caries experience in overall population. Thus, to the best of our knowledge, the findings of the present systematic review show for the first time that there is a significant association of some socioeconomic indicators, like geographical area and the GNI World Bank index with ECC prevalence and experience worldwide in the last 10 years. As regards to the geographical area, there was a significant association between high caries prevalence and region of Asia and Oceania [36]. At the same time within the Asian continent, both the absolute highest and the lowest prevalence were reported, namely for the Philippines and Japan [18, 29], respectively. These findings suggest considerable inequalities in caries burden in countries with very different levels of oral healthcare awareness, dietary choices, national healthcare systems and income.

Especially as regards GNI, an inverse association with both ECC prevalence and experience was observed. As lower GNI of the country indicated higher caries prevalence and experience, it can be speculated that current caries prevention consensus papers should consider the socioeconomic climate, when designing prevention programs. On the contrary, the results of the study revealed no significant statistical correlation between Gini coefficient and caries prevalence/experience. In fact, contrary to the expectations, the analysis showed higher prevalence was observed in those countries with no and medium inequalities. This suggests that existing health care systems in low inequality countries do not solve the problem of persisting high ECC prevalence, but rather enhance existing inequalities due to the financial incentives towards more invasive treatment options (restorative therapy).

Early childhood caries negatively impacts the oral health-related quality of life for preschool children and their parents. The life expectancy at birth is a widely used metric to assess health condition. An increased standard of living, a better lifestyle, more education, and easier access to high-quality healthcare are some of the reasons for the increases in life expectancy at birth. Countries with the highest life expectancy (> 80 years) presented the lowest pooled mean dmft = 1.34 teeth while the highest dmft (5.18 teeth) was found in countries with a life expectancy between 71 and 75 years as well as ECC prevalence ranged from 28% in countries with the highest life expectancy (< 80 years) to 62% in countries with lower (71-75-year) life expectancy. These findings are in line with the body of research emphasizing how crucial it is to evaluate socioeconomic circumstances in addition to clinical and OHRQoL assessments, especially in preschoolers.

The study has several limitations, such as lack of data on ECC prevalence and experience for several countries. While many publications were available from the Asian continent mostly from countries like China and India, very few data were available from the African continent. Only 6 African countries reported relevant data matching the search criteria of the review. However, since those six countries are well-distributed throughout the continent, they may at least give a general estimation of the ECC prevalence in this geographical area. It is also important to bear in mind, that ECC data was not available for all the countries, which clearly influences the overall estimation for the prevalence worldwide.

Furthermore, as the data in the studies were collected in regional or local populations, the results might poorly represent the overall situation on a country level. High heterogeneity was reported between studies. Although heterogeneity could be due to the nature of proportional data, true heterogeneity is expected in prevalence estimates due to differences in the time and place where included studies were conducted [37]. In the light of the aforementioned limitations, results should be interpreted with caution.

Included studies show that a wide variation in ECC prevalence exists across geographical areas and remain high. More standardized and regular research is needed to monitor the oral health situation on an individual (caregiver/infant) level, as there is a clear link between health oral habits and socioeconomic challenges within the communities. This highlights the need for further research into socioeconomic indicators within the context of the cultural and socioeconomic differences. The challenges of persisting high caries prevalence may be resolved through addressing the needs of the caregivers within community, since the success of ECC prevention programs depends on the caregivers who are responsible for safeguarding the oral health of toddlers. In summary, the present paper evaluates for the first time the associations between global early childhood caries and socioeconomic indicators (geographical area, gross national income, inequality distribution). As the prevalence of early childhood caries remains high despite the implementation of numerous prevention programs, the findings of the present systematic review show that there is a demand to modify those programs with a clear understanding of the individual demands within the community and its socioeconomic realities. The paper outlines the rationale behind the demand for standardized and regular research in order to monitor and address the existing oral health issues, and its connection to the individual socioeconomic challenges within each country or even a region. It is of a paramount importance for researchers in the field of pediatric dentistry to have a clear understanding of the links that exist between socioeconomic indicators and oral health problems, in order to better address weaknesses of currently implemented programs. This will help to re-direct the focus of research strategies in the direction of personalized and society-based approach in order to create more effective preventive programs to reduce early childhood caries prevalence and experience worldwide.

### Electronic supplementary material

Below is the link to the electronic supplementary material.

Supplementary Material 1

## Data Availability

Data is provided within the manuscript or supplementary information files.

## Abbreviations

PRISMA: Preferred Reporting Items for Systematic Reviews and Meta-Analyses
GNI: Gross national income
Gini-index: Inequality index
WHO: World health organization
dmft: Caries experience in the primary dentition
dt: Decayed teeth in the primary dentition
mt: Missing teeth in the primary dentition
ft: Filled teeth in the primary dentition
NR: Not reported
SD: Standard deviation
