# Supplementary material for: A systematic review and meta-analysis on early-childhood-caries global data
Source: BMC Oral Health. 2024 Jul 24;24:835. doi: 10.1186/s12903-024-04605-y (PMC11267837; doi:10.1186/s12903-024-04605-y)
Supplement: Supplementary file 1 — Supplementary Material 1 [file 12903_2024_4605_MOESM1_ESM.docx]

**SUPPLEMENTARY MATERIAL**

**“A systematic review and Meta-analyses on Early-Childhood-Caries global data”**

**Content**

[Appendix Table 1. Search strategy for each electronic data base 2](#_Toc121389589)

[Appendix Table 2. Prisma 2020 check list 3](#_Toc121389589)

[Appendix Table 3. List of excluded studies (Total n=266). 5](#_Toc121389590)

[Appendix Table 4. Quality assessment of the included papers 6](#_Toc121389591)

[Appendix Table 5. Overall data of caries prevalence and experience 10](#_Toc121389592)

[Appendix Table 6. Bivariate analysis (association) between ECC prevalence categories and socioeconomic indicators. 13](#_Toc121389593)

[Appendix Table 7. Bivariate analysis (association) between ECC experience categories and socioeconomic indicators. 14](#_Toc121389594)

Appendix Table 1. Search strategy for each electronic data base**.**

| ***PubMed*** | ((((Caries) AND (Early Childhood Caries)) OR ((toddler)) AND (preschool children))) AND ((Epidemiology) OR (Survey)) |
| --- | --- |
| ***Embase*** | (('caries'/exp OR caries) AND ('early childhood caries'/exp OR 'early childhood caries' OR (early AND ('childhood'/exp OR childhood) AND ('caries'/exp OR caries))) OR 'toddler'/exp OR toddler) AND ('preschool children'/exp OR 'preschool children' OR (('preschool'/exp OR preschool) AND ('children'/exp OR children))) AND ('epidemiology'/exp OR epidemiology) |
| ***Web of Science*** | ((((Caries) AND (Early Childhood Caries)) OR ((toddler)) AND (preschool children))) AND (Epidemiology) OR (Survey)) |
| ***Open Grey*** | Caries AND Early Childhood Caries |

Appendix Table 2. Prisma 2020 check list

| **Section and Topic** | **Item #** | **Checklist item** | **Location where item is reported** |
| --- | --- | --- | --- |
| **TITLE** | | |  |
| Title | 1 | Identify the report as a systematic review. | 1 |
| **ABSTRACT** | | |  |
| Abstract | 2 | See the PRISMA 2020 for Abstracts checklist. | 2 |
| **INTRODUCTION** | | |  |
| Rationale | 3 | Describe the rationale for the review in the context of existing knowledge. | 3 |
| Objectives | 4 | Provide an explicit statement of the objective(s) or question(s) the review addresses. | 3 |
| **METHODS** | | |  |
| Eligibility criteria | 5 | Specify the inclusion and exclusion criteria for the review and how studies were grouped for the syntheses. | 4,5 |
| Information sources | 6 | Specify all databases, registers, websites, organisations, reference lists and other sources searched or consulted to identify studies. Specify the date when each source was last searched or consulted. | 4 |
| Search strategy | 7 | Present the full search strategies for all databases, registers and websites, including any filters and limits used. | 4 |
| Selection process | 8 | Specify the methods used to decide whether a study met the inclusion criteria of the review, including how many reviewers screened each record and each report retrieved, whether they worked independently, and if applicable, details of automation tools used in the process. | 4,5 |
| Data collection process | 9 | Specify the methods used to collect data from reports, including how many reviewers collected data from each report, whether they worked independently, any processes for obtaining or confirming data from study investigators, and if applicable, details of automation tools used in the process. | 5 |
| Data items | 10a | List and define all outcomes for which data were sought. Specify whether all results that were compatible with each outcome domain in each study were sought (e.g. for all measures, time points, analyses), and if not, the methods used to decide which results to collect. | 5 |
|  | 10b | List and define all other variables for which data were sought (e.g. participant and intervention characteristics, funding sources). Describe any assumptions made about any missing or unclear information. | 6 |
| Study risk of bias assessment | 11 | Specify the methods used to assess risk of bias in the included studies, including details of the tool(s) used, how many reviewers assessed each study and whether they worked independently, and if applicable, details of automation tools used in the process. | 5 |
| Effect measures | 12 | Specify for each outcome the effect measure(s) (e.g. risk ratio, mean difference) used in the synthesis or presentation of results. | 5,6 |
| Synthesis methods | 13a | Describe the processes used to decide which studies were eligible for each synthesis (e.g. tabulating the study intervention characteristics and comparing against the planned groups for each synthesis (item #5)). | 5 |
|  | 13b | Describe any methods required to prepare the data for presentation or synthesis, such as handling of missing summary statistics, or data conversions. | 5 |
|  | 13c | Describe any methods used to tabulate or visually display results of individual studies and syntheses. | 5 |
|  | 13d | Describe any methods used to synthesize results and provide a rationale for the choice(s). If meta-analysis was performed, describe the model(s), method(s) to identify the presence and extent of statistical heterogeneity, and software package(s) used. | 5 |
|  | 13e | Describe any methods used to explore possible causes of heterogeneity among study results (e.g. subgroup analysis, meta-regression). | 5 |
|  | 13f | Describe any sensitivity analyses conducted to assess robustness of the synthesized results. | 5,6 |
| Reporting bias assessment | 14 | Describe any methods used to assess risk of bias due to missing results in a synthesis (arising from reporting biases). | 5,6 |
| Certainty assessment | 15 | Describe any methods used to assess certainty (or confidence) in the body of evidence for an outcome. | 5,6 |
| **RESULTS** | | |  |
| Study selection | 16a | Describe the results of the search and selection process, from the number of records identified in the search to the number of studies included in the review, ideally using a flow diagram. | 7 |
|  | 16b | Cite studies that might appear to meet the inclusion criteria, but which were excluded, and explain why they were excluded. | 7 |
| Study characteristics | 17 | Cite each included study and present its characteristics. | 7 |
| Risk of bias in studies | 18 | Present assessments of risk of bias for each included study. | 8 |
| Results of individual studies | 19 | For all outcomes, present, for each study: (a) summary statistics for each group (where appropriate) and (b) an effect estimate and its precision (e.g. confidence/credible interval), ideally using structured tables or plots. | 8 |
| Results of syntheses | 20a | For each synthesis, briefly summarise the characteristics and risk of bias among contributing studies. | 8,9 |
|  | 20b | Present results of all statistical syntheses conducted. If meta-analysis was done, present for each the summary estimate and its precision (e.g. confidence/credible interval) and measures of statistical heterogeneity. If comparing groups, describe the direction of the effect. | 8,9 |
|  | 20c | Present results of all investigations of possible causes of heterogeneity among study results. | 8 |
|  | 20d | Present results of all sensitivity analyses conducted to assess the robustness of the synthesized results. | 8 |
| Reporting biases | 21 | Present assessments of risk of bias due to missing results (arising from reporting biases) for each synthesis assessed. | 8,9 |
| Certainty of evidence | 22 | Present assessments of certainty (or confidence) in the body of evidence for each outcome assessed. | 8,9 |
| **DISCUSSION** | | |  |
| Discussion | 23a | Provide a general interpretation of the results in the context of other evidence. | 10 |
|  | 23b | Discuss any limitations of the evidence included in the review. | 11 |
|  | 23c | Discuss any limitations of the review processes used. | 11 |
|  | 23d | Discuss implications of the results for practice, policy, and future research. | 11,12 |
| **OTHER INFORMATION** | | |  |
| Registration and protocol | 24a | Provide registration information for the review, including register name and registration number, or state that the review was not registered. | 3 |
|  | 24b | Indicate where the review protocol can be accessed, or state that a protocol was not prepared. | 3 |
|  | 24c | Describe and explain any amendments to information provided at registration or in the protocol. | 3 |
| Support | 25 | Describe sources of financial or non-financial support for the review, and the role of the funders or sponsors in the review. | 13 |
| Competing interests | 26 | Declare any competing interests of review authors. | 13 |
| Availability of data, code and other materials | 27 | Report which of the following are publicly available and where they can be found: template data collection forms; data extracted from included studies; data used for all analyses; analytic code; any other materials used in the review. | 13 |

# Appendix Table 3. List of excluded studies (Total n=266).

| **Reason for exclusion 1 – no dmft data available for (n=185)** | Akinkugbe, 2021; Achmad; 2018, Achmad; 2019, Al-Sane, 2021; Akinkugbe, 2021; Alade, 2021; Amin, 2015; Abedizadeh, 2021; Arrow, Klobas, 2015; Arora, 2011; BaniHani, 2020; Bartosova, 2019; Berkowitz, 2011; Bittencourt, 2020; Boustedt, 2020; Boustedt, 2020; Berkowitz, 2011; Bulani, 2020; Broughton, 2013; Buckeridge, 2021; Campos, 2019; Carvalho, 2011; Chedid, 2012; Canares, 2018; Chaffe, 2017; Chaffe, 2014; Chandna, 2018; Chen, 2018; Cho, 2020; Colombo 2019; Chedid, 2012; Cildir, 2012; Clovis, 2012; Colvara, 2021; Costa 2018; Culler, 2021; Daher, 2014; Da Silva, 2020; De Paula, 2011; Dharmani, 2019;Díaz, 2018; Do, 2014; dos Santos Junior, 2014; Dursun, 2016; Duverger, 2014; Edelstein, 2016; El Batawi, 2014; Evans, 2013; Evans, 2018; Faria, 2020; Folayan, 2019; Folayan,Alade 2020; Folayan, Alimi 2020; Folayan, El Tantawi 2020; Folayan, El Tantawi, Vukovic, 2020; Folayan, Oginni, 2020; Folayan, 2019; Fontana, 2018; Fung, 2016; Gaidhane, 2013; Gandeeban, 2016; Gao, 2020; Gaffar, 2020; Gudipaneni, 2019; Guedes, 2014; Guedes, 2020; Gussy, 2020; Ghazal, 2015; George, 2018; Haag, 2019; Hajianpour, 2017; Hallas, 2011; Hariyani, 2020; Hashim, 2011; Heaton, 2017; Henry, 2017; Hu, 2019; Horowitz, 2017; Hong, 2014; Horowitz, 2017; Hurley, 2019; Ivančević, 2015; Jackson, 2018; Javed, 2017; Jayakaran, 2020; Jordan, 2016; Jurczak, 2015; Kato, 2015; Kakade, 2011; Kelly, 2019; Koh, 2015; Lenčová, 2012; Levine, 2012; Lin, 2019; Masumo, 2014; Manthur, 2014; Maulitasari, 2020; Meurman, 2011; Milgrom, 2013; Mishra, 2013; Míšová, 2016; Mitrakul, 2013; Moimaz, 2014; Mota, 2013; Muraleedhar Seetha, 2020; Muller-Bolla, 2017; Nahas, 2020; Naidu, 2016; Naidu, 2020; Neves, 2016; Ng, 2012; Ng, 2016; Nishide, 2018; Nicole, 2014; Nicol, 2015; Ozer, 2011; Parisotto, 2012; Parisotto, 2015; Paglia, 2016; Peerbhay, 2012; Peltzer, 2014; Pesaressi,2014; Petrauskienė, 2020; Phantumvanit, 2018; Pine 2016; Piva, 2017; Plonka 2012; Priyadarshini, 2017; Purnima, 2019; Ramos-Jorge, 2014; Rataj-Kulmacz, 2018; Renugalakshmi, 2021; Reed, 2020; Ribeiro, 2017; Rubin, 2019; Saleem, 2015; Samnaliev, 2015; Sankeshwari 2012; Sayyed, 2014; Saraithong, 2015; Schluter, 2020; Shackleton, 2018; Schroth, 2015; Schroth, 2014; Schüttfort, 2020; Scorca, 2020; Setiawan, 2020; Shimpi, 2021; Sevekar, 2018; Sharna, 2019; Shukri, 2020; Slusar, 2016; Smith, 2021; Tanaka, 2013; Targino, 2011; Thomson, 2014; Tiwari, 2014; Tušek, 2012; Tiwari, 2014; Togoo, 2016; Tong, 2014; Tonpe, 2019; Tschammler, 2018; Un Lam, 2017; Vania, 2011; Virtanen, 2021; Wagner, 2017; Wagner, 2020; Wang, 2016; Weber-Gasparoni, 2013; Weber-Gasparoni, 2013; Widyarman, 2021; Wigen, 2014; Wilson, 2018; Winter, 2019; Xiao, 2018; Yokoi, 2020; Yoon, 2012; Zaki, 2015; Zaror, 2020; Zhang, 2018; Zhan, 2012; Zhou, 2011; Zhou, 2012; Zhu, 2019; |
| --- | --- |
| **Reason for exclusion 2 - RCTs (n=32)** | Agouropoulos, 2014; Arrow, 2016; Arrow, 2015; Arrow, Klobas, 2017; Batliner, 2014; Blinkhorn, 2012; Blue, 2020; Chaffe, 2013; Colvara, 2018; Gao, 2020; Gao, 2021; Garcia, 2017; Goyal, 2019; Hedayati-Hajikand, 2015; Ismail, 2011; Jiang, 2014; Jiang, 2020; Jordan, 2017; Lotto, 2020; Mabangkhru, 2020; Maguire, 2020; Memarpour,  2016; Mohebbi, 2012; Muller-Bolla, 2018; Muñoz-Millán, 2018; Naidu, 2015; Piwat, 2020; Plonka, 2013; Plutzer 2012; Pukallus, 2013; Samuel, 2020; Thomas, 2016 |
| **Reason for exclusion 3 - CCs (n=21)** | Agarwal, 2011; Alade, 2021; Arrow, Klobas, 2016; Bimstein, 2018; Blinkhorn, 2013; Dabawala, 2017; Fan, 2016; Ismail, 2018; Iyun, 2014; Jayakaran, 2020; Kopycka-Kedzierawski, 2013; Achan, 2020; Mahesh, 2013; Nembhwani, 2020; Plonka, 2013; Plonka 2013; Plutzer, 2011; Qin, 2012; Samaddar,2021; Schüler, 2018; Smitt, 2018 |
| **Reason for exclusion 4 – Studies conducted earlier than 2011 (n=12)** | Dabiri, 2016; Dudding, 2015; Han, 2014; Hoffmeister, 2016; Hsieh, 2014; Jordan, 2020; Kopycka-Kedzierawski, 2011; Olak, 2012; Pieper, 2012; Ribeiro, 2014; Schroth, 2013; Wigen, 2018 |
| **Reason for exclusion 5 – Age group/data from patients with disabilities and/or severe health issues (n=11)** | Cianetti, 2016; de Sousa, 2021; Duijster, 2014; Laitala, 2013; Mischu, 2018; Moralez-Chávez, 2017; Mothupi, 2016; Proc, 2019; Rajonson, 2017; Sagheri, 2013 ; Smith, 2021; |
| **Reason for exclusion 6 – Excluded during further evaluation of the papers due to lack of inclusion criteria and relevant information (n=5)** | Collado, 2017; Kierce, 2016; Kraljevic, 2017; Ma, 2015; Xiao, 2018; |

Appendix Table 4. Quality assessment of the included papers (tool developed by The National Heart, Lung and Blood Institute for Observational Cohort and Cross-sectional studies, Case-Control studies and Controlled-Intervention studies (<https://www.nhlbi.nih.gov/health-topics/study-quality-assessment-tools>).

| Study ID | Title | Source | Country | Area | Quality Assessment |
| --- | --- | --- | --- | --- | --- |
| Hysi et al., 2017 | Dental caries experience among Albanian pre-school children: a national survey | Community Dental Health | Albania | Europe | fair |
| Gibbs et al., 2016 | Child oral health in migrant families: A cross-sectional study of caries in 1-4 year old children from migrant backgrounds residing in Melbourne, Australia | Community dental health | Australia | Asia/Oceania | fair |
| Parisotto et al., 2012 | Assessment of cavitated and active non-cavitated caries lesions in 3- to 4-year-old preschool children: A field study | International Journal of Paediatric Dentistry | Brazil | South America | low |
| Moimaz et al., 2016 | Early Childhood Caries: Epidemiology, Severity and Sociobehavioural Determinants | Oral health & preventive dentistry | Brazil | South America | fair |
| Ferraz et al., 2014 | Clinical consequences of untreated dental caries and toothache in preschool children | Pediatric dentistry | Brazil | South America | low |
| Pinto et al., 2017 | Are Maternal Factors Predictors for Early Childhood Caries? Results from a Cohort in Southern Brazil | Brazilian dental journal | Brazil | South America | fair |
| Antunes et al., 2018 | Oral health outcomes: the association of clinical and socio-dental indicators to evaluate dental caries in preschool children | Ciência & Saúde Coletiva | Brazil | South America | fair |
| Colombo et al., 2017 | Quantitative assessment of salivary oral bacteria according to the severity of dental caries in childhood | Archives of Oral Biology | Brazil | South America | fair |
| Kubota et al., 2020 | Association between Early Childhood Caries and Maternal Factors among 18- to 36-month-old Children in a Rural Area of Cambodia | Oral health & preventive dentistry | Cambodia | Asia/Oceania | fair |
| Turton et al., 2019 | Epidemiological survey of early childhood caries in Cambodia | BMC oral health | Cambodia | Asia/Oceania | fair |
| Turton et al., 2016 | Socio-behavioural risk factors for early childhood caries (ECC) in Cambodian preschool children: a pilot study | European Archives of Paediatric Dentistry | Cambodia | Asia/Oceania | fair |
| Kubota et al., 2020 | Early childhood caries status and its associated factors among young children in a rural area of Cambodia | Pediatric Dental Journal | Cambodia | Asia/Oceania | fair |
| Azrak et al., 2017 | The Oral Health of Preschool Children of Refugee and Immigrant Families in Manitoba | J Can Dent Assoc | Canada | Asia/Oceania | fair |
| Zhu et al., 2020 | Early childhood caries and related risk factors: A cross-sectional study of children in Zhejiang Province, China | International journal of dental hygiene | China | Asia/Oceania | fair |
| Sun et al., 2020 | The association between postpartum depression and early childhood caries | Acta odontologica Scandinavica | China | Asia/Oceania | low |
| Wu et al., 2020 | Risk factors of dental caries among young children in Pudong New District, Shanghai | Shanghai journal of stomatology | China | Asia/Oceania | low |
| Wang et al., 2017 | A longitudinal study of early childhood caries incidence in Wenzhou preschool children | BMC oral health | China | Asia/Oceania | fair |
| Li et al., 2017 | Prevalence of severe early childhood caries and associated socioeconomic and behavioral factors in Xinjiang, China: a cross-sectional study | BMC oral health | China | Asia/Oceania | fair |
| Zhang et al., 2020 | Assessment of risk factors for early childhood caries at different ages in Shandong, China and reflections on oral health education: a cross-sectional study | BMC oral health | China | Asia/Oceania | fair |
| Bao et al., 2014 | Prevalence of severe early childhood caries and associated socioeconomic and behavioral factors in Xinjiang, China: a cross-sectional study | BMC Oral Health | China | Asia/Oceania | fair |
| Jiang et al., 2017 | Prevalence of Early Childhood Caries Among 2- to 5-year-old Preschoolers in Kindergartens of Weifang City, China: A Cross-sectional Study | Oral health & preventive dentistry | China | Asia/Oceania | low |
| Li et al., 2020 | The status and associated factors of early childhood caries among 3- to 5-year-old children in Guangdong, Southern China: a provincial cross-sectional survey | BMC oral health | China | Asia/Oceania | low |
| Wulaerhan et al., 2014 | Risk determinants associated with early childhood caries in Uygur children: a preschool-based cross-sectional study | BMC oral health | China | Asia/Oceania | fair |
| Gavic et al., 2015 | The role of parental anxiety, depression, and psychological stress level on the development of early-childhood caries in children | International journal of paediatric dentistry | Croatia | Europe | fair |
| Acuña et al., 2019 | Prevalence of Early Childhood Caries in Children Aged 1 to 5 Years in the City of Quito, Ecuador | The Open Dentistry Journal | Ecuador | South America | low |
| Tubert-Jeannin et al., 2012 | Addressing children's oral health inequalities: caries experience before and after the implementation of an oral health promotion program | Acta Odontologica Scandinavica | France | Europe | low |
| Bourgeois et al., 2014 | Global burden of dental condition among children in nine countries participating in an international oral health promotion programme, 2012-2013 | International dental journal | France | Europe | fair |
| Deichselet al., 2021 | Frühkindliche Karies und assoziierte  Risikofaktoren bei Kleinkindern  im Land Brandenburg | Bundesgesundheitsblat | Germany | Europe | low |
| Meyer et al., 2017 | Sociodemographic determinants of spatial disparities in early childhood caries: An ecological analysis in Braunschweig, Germany | Community dentistry and oral epidemiology | Germany | Europe | fair |
| Boka et al., 2013 | Dental caries and oral health-related factors in a sample of Greek preschool children | Eur Arch Paediatr Dent | Greece | Europe | low |
| Pikramenou et al., 2016 | Association between dental caries and body mass in preschool children | European Archives of Paediatric Dentistry | Greece | Europe | low |
| Wong et al., 2011 | Oral health-related quality of life in Hong Kong preschool children | Caries Research | Hong Kong | Asia/Oceania | fair |
| Gao et al., 2018 | Risk Factors of Early Childhood Caries among Young Children in Hong Kong: A Cross-Sectional Study | The Journal of clinical pediatric dentistry | Hong Kong | Asia/Oceania | low |
| Duangthip et al., 2020 | Oral health-related quality of life and caries experience of Hong Kong preschool children | International Dental Journal | Hong Kong | Asia/Oceania | fair |
| Gao et al., 2013 | Validity of caries risk assessment programmes in preschool childre | Journal of dentistry | Hong Kong | Asia/Oceania | fair |
| Prakasha Shrutha et al., 2013 | Feeding practices and early childhood caries: A cross-sectional study of preschool children in Kanpur District, India | ISRN Dentistry | India | Asia/Oceania | fair |
| Barjatya et al., 2020 | Association between early childhood caries and feeding practices among 3-5-year-old children of Indore, India | Journal of Indian Society of Pedodontics and Preventive Dentistry | India | Asia/Oceania | low |
| Kakanur et al., 2017 | Exploring the multitude of risk factors associated with early childhood caries | Indian journal of dental research | India | Asia/Oceania | low |
| Jain et al., 2018 | Sociodemographic and behavioral factors associated with early childhood caries among preschool children of Western Maharashtra | Indian journal of dental research | India | Asia/Oceania | low |
| Sharma et al., 2019 | A cross-sectional study to assess the prevalence of early childhood caries and associated risk factors in preschool children in district Mandi, Himachal Pradesh | Journal of Indian Society of Pedodontics and Preventive Dentistry | India | Asia/Oceania | low |
| Sankeshwari et al., 2013  Schultz, 2022 | Association of socio-economic status and dietary habits with early childhood caries among 3- to 5-year-old children of Belgaum city  Kariespravalenz und Karieserfahrung bei 3- bis 6 - jarigen Kindern in Hamburg | Oralprophylaxe Kinderzahnhelkd  European archives of paediatric dentistry | India | Asia/Oceania | fair |
| Nagarajappa et al., 2020 | Feeding practices and early childhood caries among preschool children of Bhubaneswar, India | European Archives of Paediatric Dentistry | India | Asia/Oceania | fair |
| Gopal et al., 2016 | Prevalence and predictors of early childhood caries in 3- to 6-year-old south Indian children - A crosssectional descriptive study | Oral Health and Preventive Dentistry | India | Asia/Oceania | low |
| Bagherian et al.,   2013 | Association between dental caries and age-specific body mass index in preschool children of an Iranian population | Indian J Dent Res | Iran | Asia/Oceania | fair |
| Rashid Noaman et al., 2019 | Maternal dental health knowledge and its relation to the dental caries experience of their children in mamyzawa camp of refugees in Erbil, Iraq | Acta Medica Academica | Iraq | Asia/Oceania | fair |
| Nobile et al., 2014 | Pattern and severity of early childhood caries in Southern Italy: a preschool-based cross-sectional study | BMC public health | Italy | Europe | fair |
| Severino et al.,2021 | Prevalence of Early Childhood Caries (ECC) in a paediatric italian population: An epidemiological study | European journal of paediatric dentistry | Italy | Europe | low |
| Congiu et al., 2014 | Early childhood caries and associated determinants: a cross-sectional study on Italian preschool children | Journal of public health dentistry | Italy | Europe | fair |
| Nakayama et al., 2015 | Association of environmental tobacco smoke and snacking habits with the risk of early childhood caries among 3-year-old Japanese children | Journal of Public Health Dentistry | Japan | Asia/Oceania | fair |
| Nakayama et al., 2019 | Association of Environmental Tobacco Smoke with the Risk of Severe Early Childhood Caries among 3-Year-Old Japanese Children | Caries research | Japan | Asia/Oceania | fair |
| Nakayama et al., 2015 | Association between nocturnal breastfeeding and snacking habits and the risk of early childhood caries in 18- to 23-month-old Japanese children | Journal of epidemiology | Japan | Asia/Oceania | fair |
| Rajab et al., 2020 | Impact of dental caries on the quality of life of preschool children and families in Amman, Jordan | Quintessence international | Jordan | Asia/Oceania | fair |
| Ozen et al., 2016 | Evaluation of Possible Associated Factors for Early Childhood Caries and Severe Early Childhood Caries: A Multicenter Cross-Sectional Survey | The Journal of clinical pediatric dentistry | Netherlands | Europe | fair |
| Folayan et al., 2015 | Prevalence, and early childhood caries risk indicators in preschool children in suburban Nigeria | BMC oral health | Nigeria | Africa | fair |
| Olatosi et al., 2021 | Replication of GWAS significant loci in a sub-Saharan African Cohort with early childhood caries: a pilot study | BMC oral health 2021 | Nigeria | Africa | fair |
| Olczak-Kowalczyk et al., 2020 | Factors associated with early childhood caries in Polish three-year-old children | Oral Health and Preventive Dentistry | Poland | Europe | fair |
| Pereira et al., 2021 | Prevalence and sociobehavioural determinants of early childhood caries among 5-year-old Portuguese children: a longitudinal study | European archives of paediatric dentistry | Portugal | Europe | fair |
| Alkhtib et al., 2016 | Prevalence of early childhood caries and enamel defects in four and five-year old  Qatari preschool children | BMC Oral Health | Qatar | Asia/Oceania | fair |
| Igic et al., 2018 | Prevalence and progression of early childhood caries in Niš, Serbia | European Journal of Paediatric Dentistry | Serbia | Europe | low |
| Tušek et al., 2020 | Early childhood caries in multilingual community | Central European journal of public health | Serbia | Europe | low |
| Perera et al., 2014 | Effect of feeding practices on dental caries among preschool children: A hospital based analytical cross sectional study | Asia Pacific Journal of Clinical Nutrition | Sri Lanka | Asia/Oceania | low |
| Baggio et al., 2015 | Early childhood caries in Switzerland: a marker of social inequalities | BMC Oral Health | Switzerland | Europe | fair |
| Qadri et al., 2012 | Early childhood caries and feeding practices in kindergarten children | Quintessence international | Syria | Asia/Oceania | fair |
| Ndekero et al., 2021 | Prevalence of early childhood caries, risk factors and nutritional status among 3-5-yearold preschool children in Kisarawe, Tanzania | PLoS ONE | Tanzania | Africa | fair |
| Peltzer et al., 2015 | Severe early childhood caries and social determinants in three-year-old children from Northern Thailand: a birth cohort study | BMC oral health | Thailand | Asia/Oceania | fair |
| Leelataweewud et al., 2021 | Psychometric evaluation of the Thai version of the Early Childhood Oral Health Impact Scale (Th-ECOHIS): a cross sectional validation study | BMC oral health | Thailand | Asia/Oceania | low |
| Pattanaporn et al., 2013 | Mode of delivery, mutans streptococci colonization, and early childhood caries in three- to five-year-old Thai children | Community Dentistry and Oral Epidemiology | Thailand | Asia/Oceania | fair |
| Naidu et al., 2016 | Oral health-related quality of life and early childhood caries among preschool children in Trinidad | BMC Oral Health | Trinidad | South America | fair |
| Naidu et al., 2013 | Socio-behavioural factors and early childhood caries: a cross-sectional study of preschool children in central Trinidad | BMC oral health | Trinidad | South America | fair |
| Elelmi et al., 2021 | Black stain and dental caries in primary teeth of Tunisian preschool children | European archives of paediatric dentistry | Tunisia | Africa | fair |
| Abbasoğlu et al., 2015 | Early Childhood Caries Is Associated with Genetic Variants in Enamel Formation and Immune Response Genes | Caries Research | Turkey | Asia/Oceania | fair |
| Ozler et al., 2018 | Pufa index and related factors among 36- to 71-month-old children in Turkey: A cross-sectional study | Oral Health and Preventive Dentistry | Turkey | Asia/Oceania | low |
| Kowash et al., 2015 | Severity of early childhood caries in preschool children attending Al-Ain Dental Centre, United Arab Emirates | European archives of paediatric dentistry | UAE | Asia/Oceania | fair |
| Angelopoulou et al., 2019 | Association of food insecurity with early childhood caries | Journal of Public Health Dentistry | USA | North America | fair |
| Ghazal et al., 2015 | Prevalence and incidence of early childhood caries among African-American children in Alabama | Journal of Public Health Dentistry | USA | North America | fair |
| Warren et al., 2016 | Factors associated with dental caries in a group of American Indian children at age 36 months | Community dentistry and oral epidemiology | USA | North America | low |
| Divaris et al., 2020 | Cohort profile: Zoe 2,0—a community-based genetic epidemiologic study of early childhood oral health | International Journal of Environmental Research and Public Health | USA | North America | fair |
| Seminario et al., 2018 | Suboptimal Serum Vitamin D Associated with Early Childhood Caries in Special Health Care Needs Children | Journal of dentistry for children | USA | North America | fair |
| Huong et al., 2017 | Prevalence of early childhood caries and its related risk factors in preschoolers: Result from a cross sectional study in Vietnam | Pediatric Dental Journal | Vietnam | Asia/Oceania | fair |
| Nguen et al., 2018 | Early childhood caries and risk factors in Vietnam | Journal of Clinical Pediatric Dentistry | Vietnam | Asia/Oceania | fair |
| Khanh et al., 2015 | Early Childhood Caries, Mouth Pain, and Nutritional Threats in Vietnam | American journal of public health | Vietnam | Asia/Oceania | low |
| Boustedt, 2022 | Free sugars and early childhood caries development: a prospective | European Archives of Paediatric Dentistry | Sweden | Europe | high |
| Cortes, 2017 | Caries status in young Colombian children | Acta Odontologica Scandinavica | Colombia | South America | fair |
| Carvalho, 2014 | Oral Health Determinants and Caries | Caries Research | Brazil | South America | fair |
| Munoz, 2014 | Early childhhod caries in Peru | Frontiers in Public Health | Peru | South America | fair |
| Zambrano, 2015 | Oral Health Policies to Tackle the Burden of Early Childhood Caries: A Review of 14 Countries/Regions | Frontiers in Public Health | Venezuela | South America | fair |
| UK'sNationalDental Epidemiology Survey, 2020 | Oral Health Policies to Tackle the Burden of Early Childhood Caries: A Review of 14 Countries/Regions | UK's National  Dental Epidemiology Survey, 2022 | UK | Europe | high |
| Sitthisettapong, 2021 | Strategic Management of Early Childhood Caries in Thailand: A Critical Overview | Frontiers in Public Health | Thailand | Asia/Oceania | fair |
| Amalia, 2019 | Indonesia: Epidemiological Profiles of Early Childhood Caries | Frontiers in Public Health | Indonesia | Asia/Oceania | fair |
| El Fadl, 2019 | Assessing the early childhood caries and the asscociated determinants in a goup of preschool children: results from a national oral health survey in egypt | Egyptian Dental Journal | Egypt | Asia/Oceania | low |
| Davidian, 2021 | Analysis of prevalence, intensity and feaures of the clinical course of caries based on the data of preventive medical examination | Endodontics today | Russia | Europe | fair |
| Tsang, 2019 | Early Childhood oral health and nutrition in Urban and rural Nepal | International Journal of Enviromental Reasearch | Nepal | Asia/Oceania | fair |
| Lara, 2021 | Impact of early childhood caries severity on oral health-  related quality of life among preschool children in Mexico:  A cross-sectional study | International Journal of Paediatric Dentistry | Mexico | South America | high |
| Musinguzi, 2019 | Prevalence and Treatment Needs for Early Childhood Caries Among 3–5-Year-Old Children From a Rural Community in Uganda | Frontiers Public Health | Uganda | Africa | fair |
| Šačić, 2016 | The prevalence and severity of early childhood caries | Acta Medica Academica | Bosnia and  Herzegovina | Europe | fair |
| Shakavets, 2018 | Preventive Programs of Early Childhood Caries in Belarus | International reviews. Clinical Practice and Health | Belarus | Europe | low |
| Songa, 2022 | Analysis of the Dental Caries Epidemiological Profile | Oral Health Prev Dent | Angola | Africa | low |
| Chouchene, 2022 | Early Childhood Caries Prevalence and Associated Risk Factors in Monastir, Tunisia: A Cross-Sectional Study | Front. Public Health | Tunisia | Africa | low |

Appendix Table 5. Overall data of caries prevalence and experience**.**

| Study ID | Country | Participants *(M/F)* | Prevalence*  *range* | dmft  *mean±SD* |
| --- | --- | --- | --- | --- |
| Nakayama, 2015 | Japan | 1675(871/804) | 1 | 0.1±0.7 |
| Folayan, 2015 | Nigeria | 497(^* *^ NR) | 1 | 0.2±NR |
| Deichsel, 2012 | Germany | 10161(5249/4912) | 1 | 0.2±1.0 |
| Nobile, 2014 | Italy | 388(194/194) | 1 | 0.5±1.4 |
| Moimaz, 2016 | Brazil | 768(391/377) | 1 | 0.5±NR |
| Warren, 2016 | USA | 232(103/129) | 4 | 0.7±1.9 |
| Nakayama, 2019 | Japan | 2277(1171/1106) | 2 | 0.8±2.1 |
| UK's Nat Dent Epid Sur, 2020 | UK | 6900(NR) | 2 | 0.8±NR |
| Tušek, 2020 | Serbia | 341(191/150) | 2 | 0.9±NR |
| Chouchene, 2022 | Tunisia | 381(195/186) | 2 | 0.9±0.2 |
| Congiu, 2014 | Italy | 544(284/260) | 1 | 0.9±0.5 |
| Nakayama, 2015 | Japan | 1801(908/893) | 2 | 1.0±2.6 |
| Meyer, 2017 | Germany | 5527(NR) | NA | 1.0±NR |
| Tubert-Jeannin, 2012 | France | 478(244/234) | 2 | 1.9±2.6 |
| Pereira, 2021 | Portugal | 142(71/71) | 3 | 1.2±2.3 |
| Boka, 2013 | Greece | 804(NR) | 2 | 1.3±3.8 |
| Elelmi, 2021 | Tunisia | 393(NR) | 3 | 1.4±0.1 |
| Gao, 2013 | Hong Kong | 544(282/262) | 2 | 1.4±2.9 |
| Wong, 2011 | Hong Kong | 1261(639/622) | 2 | 1.5±3.0 |
| Lara, 2021 | Mexico | 409(189/220) | 3 | 1.6±0.55 |
| Davidian, 2021 | Russia | 502(208/294) | 3 | 1.6±10.2 |
| Leelataweewud, 2021 | Thailand | 214(107/1070 | 3 | 1.6±2.9 |
| Bourgeois,2014 | Greece | 352(NR) | 1 | 1.6±3.8 |
| Carvalho, 2014 | Brazil | 2331(1205/1306) | 3 | 1.8±0.1 |
| Perera, 2014 | Sri Lanka | 285 (138/147) | 3 | 1.8±2.6 |
| Schulz et al., 2022 | Germany | 933(NR) | 2 | 1.8±2.7 |
| Kowash, 2015 | UAE | 176(NR) | NR | 10.9±NR |
| Bourgeois,2014 | Phillipines | 557(NR) | 4 | 12.0±5.2 |
| Prakasha Shrutha, 2013 | India | 2000(974/1026) | 3 | 2±3.0 |
| Musinguzi, 2019 | Uganda | 432(230/202) | 3 | 2±3.01 |
| Seminario, 2018 | USA | 276(137/139) | 2 | 2.1±4.0 |
| Jiang, 2017 | China | 2829(1601/1228) | 3 | 2.1±2.6 |
| Songa, 2022 | Angola | 240(NR) | 3 | 2.2±2.95 |
| Azrak, 2017 | Canada | 211(114/97) | 3 | 2.2±3.8 |
| Gibbs, 2016 | Australia | 630(322/308) | 2 | 2.3±5.6 |
| Gopal, 2016 | India | 477(214/263) | 2 | 2.4±NR |
| Igic, 2018 | Serbia | 250(NR) | 3 | 2.4±NR |
| Nagarajappa, 2020 | India | 320(157/163) | 2 | 2.4±1.9 |
| Olczak-Kowalczyk, 2020 | Poland | 734(NR) | 4 | 2.4±3.5 |
| Kubota, 2020 | Cambodia | 128(69/59) | 3 | 2.5±3.1 |
| Acuña,  2019 | Ecuador | 557(298/259) | 3 | 2.5±NR |
| Ndekero, 2021 | Tanzania | 831(401/430) | 3 | 2.5±NR |
| Ferraz, 2014 | Brazil | 540(238/302) | 3 | 2.7±3.9 |
| Kubota, 2020 | Cambodia | 121(67/54) | 3 | 2.8±3.5 |
| Boustedt, 2022 | Sweden | 208(105/103) | 2 | 2.9±3.14 |
| Wang, 2017 | China | 606(315/289) | 3 | 2.9±3.7 |
| Sankeshwari, 2013 | India | 1116(663/453) | 4 | 3±3.6 |
| Ghazal 2015 | USA | 97(57/40) | 3 | 3.1±3.9 |
| Rashid Noaman, 2019 | Iraq | 79(42/37) | 4 | 3.1±4.9 |
| Zhang, 2020 | China | 1301(651/650) | 4 | 3.2±3.9 |
| Olatosi, 2021 | Nigeria | 691(347/344) | 3 | 3.3±2.6 |
| El Fadl, 2019 | Egypt | 651(322/329) | 4 | 3.5±3.6 |
| Sharma, 2019 | India | 2859(1426/1433) | 3 | 3.5±2.5 |
| Turton, 2019 | Cambodia | 3985(1985/2000) | 3 | 3.5±4.5 |
| Rajab, 2020 | Jordan | 404(209/195) | 4 | 3.6±NA |
| Parisotto, 2012 | Brazil | 351(173/178) | 3 | 3.8±4.3 |
| Wulaerhan, 2014 | China | 670(327/343) | 4 | 3.9±3.8 |
| Pattanaporn, 2013 | Thailand | 350(191/159) | 4 | 4.0±4.5 |
| Angelopoulou, 2019 | USA | 82(39/43) | 3 | 4.1±4.4 |
| Qadri, 2012 | Syria | 400(191/209) | 4 | 4.2±4.2 |
| Gao, 2018 | Hong Kong | 5167(2722/2445) | 2 | 4.3±3.4 |
| Tsang, 2019 | Nepal | 836(439/397) | 3 | 4.3±NA |
| Zhu, 2020 | China | 2700(1350/1350) | 4 | 4.3±NA |
| Li, 2020 | China | 2592(1747/845) | 4 | 4.4±NA |
| Hysi, 2017 | Albania | 2039(1063/976) | 4 | 4.4±3.8 |
| Sitthisettapong, 2021 | Thailand | 8308(NR) | 4 | 4.5±NA |
| Wu, 2020 | China | 240(NR) | 4 | 4.5±4.9 |
| Shakavets, 2018 | Belarus | 52(NR) | 4 | 4.6±4.16 |
| Gavic, 2018 | Croatia | 235(119/116) | NA | 4.6±NA |
| Ozler, 2018 | Turkey | 729(404/325) | 4 | 4.8±4.7 |
| Bourgeois,2014 | Morocco | 272(NR) | 2 | 5±5.5 |
| Abbasoğlu, 2015 | Turkey | 259(129/130) | 3 | 5.2±5.5 |
| Barjatya, 2020 | India | 640(349/291) | 4 | 5.2 ±NA |
| Turton, 2016 | Cambodia | 362(NR) | 4 | 5.3±5.4 |
| Cortes, 2017 | Colombia | 600(NR) | 3 | 5.5±9.0 |
| Li, 2017 | China | 1727(899/828) | 4 | 5.6±3.6 |
| Kakanur, 2017 | India | 1152(NR) | 2 | 5.8±8.9 |
| Khanh, 2015 | Vietnam | 566(297/269) | 4 | 5.9±5.4 |
| Amalia, 2019 | Indonesia | 1872(NR) | 4 | 6.2±NA |
| Huong, 2017 | Vietnam | 369(194/175) | 4 | 6.7±4.7 |
| Šačić, 2016 | Bosnia Herz. | 165(80/85) | 4 | 6.8±5.25 |
| Alkhtib, 2016 | Qatar | 250(127/123) | 4 | 7.6±5.2 |
| Ozen, 2016 | Turkey | 408(216/192) | 4 | 8.0±5.1 |
| Divaris, 2020 | USA | 8057(4000/4057) | 3 | 8.0±NR |
| Bagherian, 2013 | Iran | 400(211/189) | 3 | 8.4±11.2 |
| Nguen, 2018 | Vietnam | 1028 (572/456) | 4 | 9.3±5.5 |
| Antunes, 2018 | Brazil | 446(224/222) | 2 | NR |
| Baggio, 2015 | Switzerland | 856(476/380) | 2 | NR |
| Bao, 2014 | China | 893(NR) | 4 | NR |
| Colombo, 2017 | Brazil | 136(NR) | 3 | NR |
| Duangthip,2020 | Hong Kong | 336(169/167) | 2 | NR |
| Fernandez, 2015 | Argentina | 303(NR) | 4 | NR |
| Jain, 2018 | India | 200(106/94) | 4 | NR |
| Munoz, 2014 | Peru | 212(NR) | 4 | NR |
| Naidu, 2013 | Trinidad | 251(126/125) | 2 | NR |
| Naidu, 2016 | Trinidad | 251(126/125) | 2 | NR |
| Peltzer,2015 | Thailand | 597(299/298) | 4 | NR |
| Pikramenou, 2016 | Greece | 2180(1173/1007) | 1 | NR |
| Pinto, 2017 | Brazil | 538(NR) | 1 | NR |
| Severino, 2021 | Italy | 76(44/32) | 3 | NR |
| Sun, 2020 | China | 337(44/32) | 1 | NR |
| Zambrano, 2015 | Venezuela | 293(NR) | 4 | NR |

*Caries prevalence range was calculated as follows: 1- ≤20%, 2-21-40%,3- 41-60%, 4>60%

^* *^ NR= Not Reported data

# Appendix Table 6. Bivariate analysis (association) between ECC prevalence categories and socioeconomic indicators.

***Appendix Table 6a.*** *ECC prevalence expressed as percentage (categorized as ≤20%, 21-40%, 41-60% and >60%) and geographical areas where the data were collected.*

| **Geographical Area** | **low prevalence**  *n (%)* | **medium prevalence**  *n (%)* | **Medium-high prevalence**  *n (%)* | **high prevalence**  *n (%)* |
| --- | --- | --- | --- | --- |
| **Africa** | 1 (12.50) | 2 (25.00) | 5 (62.50) | 0 (00.00) |
| **Asia and Oceania** | 2 (4.00) | 10 (20.00) | 12 (24.00) | 26 (52.00) |
| **North-America** | 0 (00.00) | 1 (16.67) | 4 (66.67) | 1 (16.67) |
| **South-America** | 2 (14.29) | 3 (21.43) | 6 (42.86) | 3 (21.43) |
| **Europe** | 5 (22.73) | 7 (31.82) | 5 (22.73) | 5 (22.73) |

*N of observations=100 Cuzick’s test for trend=-8.62±3.75 z=-2.30 exact p =0.02 (10000 Monte Carlo permutation)*

***Appendix Table 6b.*** *ECC prevalence expressed as percentage (categorized as ≤20%, 21-40%, 41-60% and >60%) and GNI index per year per capita expressed in USD.*

| **Income per year per capita (USD)** | **low prevalence**  *n (%)* | **medium prevalence**  *n (%)* | **Medium-high prevalence**  *n (%)* | **high prevalence**  *n (%)* |
| --- | --- | --- | --- | --- |
| **<5000** | 1 (3.70) | 4 (14.81) | 10 (37.04) | 12 (44.44) |
| **>5000-<10000** | 2 (12.50) | 2 (12.50) | 5 (31.25) | 7 (43.75) |
| **>10000-<20000** | 2 (9.52) | 3 (14.29) | 7 (33.33) | 9 (42.86) |
| **>20000-<40000** | 4 (26.67) | 5 (33.33) | 4 (26.67) | 2 (13.33) |
| **>40000** | 1 (4.76) | 9 (42.86) | 6 (28.57) | 5 (23.81) |

*N of observations=100 Cuzick’s test for trend=-10.69±4.12 z=-2.60 exact p<0.01 (10000 Monte Carlo permutation)*

***Appendix Table 6c.*** *ECC prevalence expressed as percentage (categorized as ≤20%, 21-40%, 41-60% and >60%) and Gini coefficient.  Gini coefficient was categorized by authors as follow: < 32 no inequalities, 32.4-35 low inequalities, 35.1- 40 medium inequalities and >40 high inequalities.*

| **Gini coefficient** | **low prevalence**  *n (%)* | **medium prevalence**  *n (%)* | **medium-high prevalence**  *n (%)* | **high prevalence**    *n (%)* |
| --- | --- | --- | --- | --- |
| **No inequalities** | 1 (11.11) | 2 (22.22) | 0 (0.00) | 6 (66.67) |
| **Low inequalities** | 3 (14.29) | 7 (33.33) | 5 (23.81) | 6 (28.57) |
| **Medium inequalities** | 4 (11.76) | 6 (17.65) | 9 (26.47) | 15 (44.12) |
| **High inequalities** | 2 (7.41) | 4 (14.81) | 15 (55.56) | 6 (22.22) |

*N of observations=91 Cuzick’s test for trend= -0.708±2.5 z=-0.3 exact p=0.08 (10000 Monte Carlo permutation)*

***Appendix Table 6d.*** *ECC prevalence expressed as percentage (categorized as ≤20%, 21-40%, 41-60% and >60%) and unemployment rate in a population in percentage (0.1 –<3.0 -low; 3.0 –<6.0 -medium; 6.0 –<10 – high and >10.0 – very high).*

| **Unemployment rate (%)** | **low prevalence**  *n (%)* | **medium prevalence**  *n (%)* | **Medium-high prevalence**  *n (%)* | **high prevalence**  *n (%)* |
| --- | --- | --- | --- | --- |
| **low** | 0 (0.00) | 2 (11.76) | 6 (35.29) | 9 (52.94) |
| **medium** | 4 (8.89) | 12 (26.67) | 13 (28.89) | 16 (35.56) |
| **high** | 0 (00.00) | 5 (31.25) | 6 (37.50) | 5 (31.25) |
| **very high** | 6 (27.27) | 4 (18.18) | 7 (31.82) | 5 (22.73) |

*N of observations=100 Cuzick’s test for trend= -6.725±2.8 z=-0.02 exact p<0.03 (10000 Monte Carlo permutation)*

***Appendix Table 6e.*** *ECC prevalence expressed as percentage (categorized as ≤20%, 21-40%, 41-60% and >60%) and life expectancy in a population in years.*

| **Life expectancy in years** | **low prevalence**  *n (%)* | **medium prevalence**  *n (%)* | **Medium-high prevalence**  *n (%)* | **high prevalence**  *n (%)* |
| --- | --- | --- | --- | --- |
| **53- <70** | 1 (8.33) | 2 (16.67) | 6 (50.00) | 3 (25.00) |
| **70-<75** | 0 (00.00) | 3 (16.67) | 7 (38.89) | 8 (44.44) |
| **75-<80** | 3 (6.67) | 5 (11.11) | 16 (35.56) | 21 (46.67) |
| **>80** | 6 (24.00) | 13 (52.00) | 3 (12.00) | 3 (12.00) |

*N of observations=100 Cuzick’s test for trend= -6.925±2.6 z=-0.01 exact p<0.01 (10000 Monte Carlo permutation)*

# Appendix Table 7. Bivariate analysis (association) between ECC experience categories and socioeconomic indicators.

***Appendix Table 7a.*** *ECC experience expressed as dmft index categorized as follows ≤ 1 (low experience), 1.01-2 (medium experience), 2.01-4 (medium-high experience) and >4 (high experience) and geographical areas where the data were collected.*

| **Geographical Area** | **low experience**  *n (%)* | **medium experience**  *n (%)* | **medium-high prevalence**  *n (%)* | **high experience**  **n (%)** |
| --- | --- | --- | --- | --- |
| **Africa** | 2 (25.00) | 1 (12.50) | 4 (50.00) | 1 (12.50) |
| **Asia and Oceania** | 3 (6.67) | 4 (8.89) | 14 (31.11) | 24 (53.33) |
| **North-America** | 1 (16.67) | 0 (00.00) | 3 (50.00) | 2 (33.33) |
| **South-America** | 1 (16.67) | 1 (16.67) | 2 (33.33) | 2 (33.33) |
| **Europe** | 6 (28.57) | 7 (33.33) | 3 (14.29) | 5 (23.81) |

*N of observations=86 Cuzick’s test for trend= -8.587±3.5 z=-0.015exact p<0.01 (10000 Monte Carlo permutation)*

***Appendix Table 7b.*** *ECC experience expressed as dmft index categorized as follows ≤ 1 (low experience), 1.01-2 (medium experience), 2.01-3,5 (medium-high experience) and >3.5 (high experience) and GNI index per year per capita expressed in USD.*

| **Income per year per capita (USD)** | **low experience**  *n (%)* | **medium experience**  *n (%)* | **medium-high experience**  *n (%)* | **high experience**  *n (%)* |
| --- | --- | --- | --- | --- |
| **<5000 USD** | 1 (4.00) | 2 (8.00) | 10 (40.00) | 12 (48.00) |
| **>5000-<10000** | 2 (18.18) | 1 (9.09) | 4 (36.36) | 4 (36.36) |
| **>10000-<20000** | 1 (6.25) | 2 (12.50) | 4 (25.00) | 9 (56.25) |
| **>20000-<40000** | 5 (35.71) | 5 (35.71) | 3 (21.43) | 1 (7.14) |
| **>40000** | 4 (20.00) | 3 (15.00) | 5 (25.00) | 8 (40.00) |

*N of observations=86 Cuzick’s test for trend= -8.273±3.9 z=-0.04 exact p=0.04 (10000 Monte Carlo permutation)*

***Appendix Table 7c.*** *ECC experience expressed as dmft index categorized as follows ≤ 1 (low experience), 1.01-2 (medium experience), 2.01-4 (medium-high experience) and >4 (high experience) and Gini coefficient. Gini coefficient was categorized by authors as follow: < 32 no inequalities, 32.4-35 low inequalities, 35.1- 40 medium inequalities and >40 high inequalities.*

| **GINI coefficient** | **low experience**  *n (%)* | **medium experience**  *n (%)* | **medium-high experience**  *n (%)* | **High experience**    *n (%)* |
| --- | --- | --- | --- | --- |
| **No inequalities** | 2 (18.18) | 1 (9.09) | 4 (36.36) | 4 (36.36) |
| **Low inequalities** | 4 (22.22) | 6 (33.33) | 2 (11.11) | 6 (33.33) |
| **Medium inequalities** | 5 (16.67) | 2 (6.67) | 10 (33.33) | 13 (43.33) |
| **High inequalities** | 2 (10.53) | 2 (10.53) | 7 (36.84) | 19 (42.11) |

*N of observations=78 Cuzick’s test for trend= -2.404±2.4 z=-0.316 exact p=0.33 (10000 Monte Carlo permutation)*

***Appendix Table 7d.*** *ECC experience expressed as dmft index categorized as follows ≤ 1 (low experience), 1.01-2 (medium experience), 2.01-4 (medium-high experience) and >4 (high experience) and unemployment rate in a population in percentage (0.1 –<3.0 -low; 3.0 –<6.0 -medium; 6.0 –<10 – high and >10.0 – very high).*

| **Unemployment rate (%)** | **low experience**  *n (%)* | **medium experience**  *n (%)* | **medium-high experience**  *n (%)* | **high experience**    *n (%)* |
| --- | --- | --- | --- | --- |
| **low** | 1 (6.67) | 1 (6.67) | 5 (33.33) | 8 (53.33) |
| **medium** | 7 (17.50) | 7 (17.50) | 13 (32.50) | 13 (32.50) |
| **high** | 0 (00.00) | 2 (16.67) | 5 (41.67) | 5 (41.67) |
| **very high** | 5 (26.32) | 3 (15.79) | 3 (15.79) | 8 (42.11) |

*N of observations=86 Cuzick’s test for trend= -2.081±2.6 z=-0.423 exact p=0.42 (10000 Monte Carlo permutation)*

***Appendix Table 7e.*** *ECC experience expressed as dmft index categorized as follows ≤ 1 (low experience), 1.01-2 (medium experience), 2.01-4 (medium-high experience) and >4 (high experience) and life expectancy in a population in years.*

| **Life expectancy in years** | **low experience**  *n (%)* | **medium experience**  *n (%)* | **medium-high prevalence**  *n (%)* | **high experience**  *n (%)* |
| --- | --- | --- | --- | --- |
| **53-<70** | 1 (9.09) | 0 (0.00) | 7 (63.64) | 3 (27.27) |
| **70-<75** | 0 (00.00) | 1 (6.67) | 7 (46.67) | 7 (46.67) |
| **75-<80** | 4 (10.53) | 5 (13.16) | 9 (23.68) | 20 (52.63) |
| **>80** | 8 (36.36) | 7 (31.82) | 3 (13.64) | 4 (18.18) |

*N of observations=86 Cuzick’s test for trend= -6.069±2.4 z=-0.01 exact p<0.01 (10000 Monte Carlo permutation)*

**Reference list of included paper into the systematic review:**

1. Abbasoʇlu Z, Tanboʇa I, Küchler E C, Deeley K, Weber M, Kaspar C. 2015. Early childhood caries is associated with genetic variants in enamel formation and immune response genes. Caries Research 49 (1): 70-77.
2. Acuña J E C, Freitas K M S, Henriques R P, Cruz E F, Binz Ordóñez M C R, Arias E C. 2019. Prevalence of early childhood caries in children aged 1 to 5 years in the city of Quito, Ecuador. Open Dentistry Journal 13(1): 242-248.
3. Alkhtib A, Ghanim A, Temple-Smith M, Messer L B, Pirotta M, Morgan M 2016. Prevalence of early childhood caries and enamel defects in four and five-year old Qatari preschool children. BMC Oral Health 16 (1): 73.
4. Amalia R, Chairunisa F, Alfian MF, Supartinah A. Indonesia: Epidemiological Profiles of Early Childhood Caries. Front Public Health. 2019 Aug 6 ;7:210. doi: 10.3389/fpubh.2019.00210. PMID: 31448251; PMCID: PMC6691044.
5. Angelopoulou M V, Shanti S D, Gonzalez C D, Love A, Chaffi J. 2019. Association of food insecurity with early childhood caries. Journal of Public Health Dentistry 79 (2): 102-108.
6. Antunes L A A, Ornellas G, Fraga R S and Antunes L S. 2018. Oral health outcomes: the association of clinical and socio-dental indicators to evaluate dental caries in preschool children. Ciencia & saude coletiva 23 (2): 491-500.
7. Azrak M E, Huang A, Hai-Santiago K, Bertone M F, DeMaré D and Schroth R J. 2017. The Oral Health of Preschool Children of Refugee and Immigrant Families in Manitoba. Journal (Canadian Dental Association) 82: h9.
8. Baggio S, Abarca M, Bodenmann P, Gehri M, Madrid C. 2015. Early childhood caries in Switzerland: A marker of social inequalities. BMC Oral Health 15(1).
9. Bagherian A, Sadeghi M, 2013. Association between dental caries and age-specific body mass index in preschool children of an Iranian population. Indian Journal of Dental Research 24(1): 66-70.
10. Bao X L, Jibek O, Yu Q Zhao J. 2014. Prevalence and risk factors for severe early childhood caries for uyghur and han children in Kashi city: A cross-sectional study. Chinese Journal of Evidence-Based Medicine 14(3): 260-264.
11. Barjatya K, Nayak U, Vatsal A, 2020. Association between early childhood caries and feeding practices among 3-5-year-old children of Indore, India. Journal of Indian Society of Pedodontics and Preventive Dentistry 38(2): 98-103.
12. Boka V, Trikaliotis A, Kotsanos N, Karagiannis V. 2013. "Dental caries and oral health-related factors in a sample of Greek preschool children." European Archives of Paediatric Dentistry 14(6): 363-368.
13. Bourgeois D M, Llodra J C. 2014. "Global burden of dental condition among children in nine countries participating in an international oral health promotion programme, 2012-2013." International Dental Journal 64: 27-34.
14. Boustedt K, Roswall J, Twetman S. Free sugars and early childhood caries development: a prospective cohort study. Eur Arch Paediatr Dent. 2022 Aug 17. doi: 10.1007/s40368-022-00745-3. Epub ahead of print. PMID: 35976492.
15. Carvalho JC, Silva EF, Vieira EO, Pollaris A, Guillet A, Mestrinho HD. Oral health determinants and caries outcome among non-privileged children. Caries Res. 2014;48(6):515-23. doi: 10.1159/000360709. Epub 2014 Jun 5. PMID: 24902889.
16. Colombo N H, Kreling P F, Ribas L F F, Pereira J A, Kressirer C A, Klein M I, et al. 2017. Quantitative assessment of salivary oral bacteria according to the severity of dental caries in childhood. Archives of Oral Biology 83: 282-288.
17. Congiu G, Campus G, Sale S, Spano G, Cagetti M G, Lugliè P F. 2014. Early childhood caries and associated determinants: a cross-sectional study on Italian preschool children. Journal of Public Health Dentistry 74(2): 147-152.
18. Cortes A, Ekstrand KR, Gamboa LF, González L, Martignon S. Caries status in young Colombian children expressed by the ICCMS™ visual/radiographic combined caries staging system. Acta Odontol Scand. 2017 Jan;75(1):12-20. doi: 10.1080/00016357.2016.1242154. Epub 2016 Oct 24. PMID: 27776449.
19. Chouchene F, Masmoudi F, Baaziz A, Maatouk F, Ghedira H. Early Childhood Caries Prevalence and Associated Risk Factors in Monastir, Tunisia: A Cross-Sectional Study. Front Public Health. 2022 Feb 25; 10:821128. doi: 10.3389/fpubh.2022.821128. PMID: 35284400; PMCID: PMC8914024.
20. Davidian O M, Fomina A V, Lukyanova E A, Shimkevich E M, Nazarova D A, Kotelnikova A P, Shilyaeva E S. Analysis of the prevalence, intensity and features of the clinical course of caries in early childhood and preschool children based on the data of preventive medical examinations. Endodontics Today. 2021;19(3):153-159. (In Russ.) https://doi.org/10.36377/1683-2981-2021-19-3-153-159
21. Deichsel M, Rojas G, Lüdecke K, Heinrich-Weltzien R. 2012. Early childhood caries and associated risk factors among infants in the German federal state of Brandenburg. Bundesgesundheitsblatt - Gesundheitsforschung - Gesundheitsschutz 55(11-12): 1504-1511.
22. Divaris K, Slade G D, Ferreira Zandona A G, Preisser J S, Ginnis J, Simancas-Pallares M A, et al. 2020. Cohort profile: Zoe 2.0—a community-based genetic epidemiologic study of early childhood oral health. International Journal of Environmental Research and Public Health 17(21): 1-16.
23. Duangthip D, Gao S S, Chen K J, Lo E C M, Chu C H. 2020. Oral health-related quality of life and caries experience of Hong Kong preschool children. International Dental Journal 70(2): 100-107.
24. El Fadl A, Khaled R et al. Assessing the prevalence of early childhood caries and the associated determinants in a group of preschool children: results from a national oral health survey in Egypt. Egyptian Dental Journal (2019): n. pag.
25. Elelmi, Y, Mabrouk R, Masmoudi F, Baaziz A, Maatouk F, Ghedira H. 2021. Black stain and dental caries in primary teeth of Tunisian preschool children. European archives of paediatric dentistry: official journal of the European Academy of Paediatric Dentistry 22(2): 235-240.
26. Fernandez C, Squazzi A, Bordoni N. (2015). Dental status and dental treatment demands in preschoolers from urban and underprivileged urban areas in Mendoza city, Argentina. Acta odontológica latinoamericana : AOL. 28. 13-21. 10.1590/S1852-48342015000100002.
27. Ferraz, N K, Nogueira L C, Pinheiro M L, Marques L S, Ramos-Jorge M L, Ramos-Jorge J. Clinical consequences of untreated dental caries and toothache in preschool children. Pediatric Dentistry 36(5): 389-392.
28. Folayan, M O, Kolawole K A, Oziegbe E O, Oyedele T, Oshomoji O V, Chukwumah N M, et al. 2015. Prevalence, and early childhood caries risk indicators in preschool children in suburban Nigeria. BMC Oral Health 15: 72.
29. Gao, S S, et al. 2018 Duangthip D, Lo E C M, Chu C H. Risk Factors of Early Childhood Caries among Young Children in Hong Kong: A Cross-Sectional Study." The Journal of clinical pediatric dentistry 42(5): 367-372.
30. Gao X, Wu I Di, Lo E C, Chu C H, Hsu C Y S, Wong M C. 2013. Validity of caries risk assessment programmes in preschool children. Journal of Dentistry 41(9): 787-795.
31. Gavic L, T, Tadin A, Mihanovic I, Gorseta K, Cigic L et al. 2018. The role of parental anxiety, depression, and psychological stress level on the development of early-childhood caries in children. International Journal of Paediatric Dentistry 28(6): 616-623.
32. General Dental Council UK. Search Registers. (2020). Available online at: https://olr.gdc-uk.org/SearchRegister/SearchResults (accessed October 6, 2022).
33. Ghazal T, Levy S M, Childers N K, Broffitt B, Cutter G, Wiener H W, et al. 2015. Prevalence and incidence of early childhood caries among African-American children in Alabama. Journal of Public Health Dentistry 75(1): 42-48.
34. Gibbs L, de Silva A M, Christian B, Gold L, Gussy M, Moore L, et al. 2016. Child oral health in migrant families: A cross-sectional study of caries in 1-4 year old children from migrant backgrounds residing in Melbourne, Australia. Community Dental Health 33(2): 100-106.
35. Gopal S, Chandrappa V, Kadidal U, Rayala C, Vegesna M. 2016. Prevalence and predictors of early childhood caries in 3- to 6-year-old south Indian children - A cross-sectional descriptive study. Oral Health and Preventive Dentistry 14(3): 267-
36. Huong D M, Hang L T T, Nhu Ngoc V T, Anh L Q, Son L H, Chu D T, et al. 2017. Prevalence of early childhood caries and its related risk factors in preschoolers: Result from a cross sectional study in Vietnam. Pediatric Dental Journal 27(2): 79-84.
37. Hysi D. 2017. Dental caries experience among Albanian pre-school children: a national survey. Community Dental Health(34): 46-49
38. Igic, M., R. Obradovic, G. Filipovic. 2018. Prevalence and progression of early childhood caries in Niš, Serbia. European Journal of Paediatric Dentistry 19(2): 161-164.
39. Jain R, Patil S, Shivakumar K M, Srinivasan S R et al. 2018. Sociodemographic and behavioral factors associated with early childhood caries among preschool children of Western Maharashtra. Indian journal of dental research: official publication of Indian Society for Dental Research 29(5): 568-574.
40. Jiang, Y. Y. 2017. Prevalence of Early Childhood Caries Among 2- to 5-year-old Preschoolers in Kindergartens of Weifang City, China: A Cross-sectional Study. Oral health & preventive dentistry 15(1): 89-97.
41. Kakanur M, Nayak M, Patil S S, Thakur R, Paul S T, Tewathia N. 2017. Exploring the multitude of risk factors associated with early childhood caries. Indian journal of dental research: official publication of Indian Society for Dental Research 28(1): 27-32.
42. Khanh L N, Ivey S L, Sokal-Gutierrez K, Barkan H, Ngo K M, Hoang H T, et al. 2015. Early Childhood Caries, Mouth Pain, and Nutritional Threats in Vietnam. American Journal of Public Health 105(12): 2510-2517.
43. Kowash M B. 2015. Severity of early childhood caries in preschool children attending Al-Ain Dental Centre, United Arab Emirates. European archives of paediatric dentistry: official journal of the European Academy of Paediatric Dentistry 16(4): 319-324.
44. Kubota Y, San Pech N, Durward C, Ogawa H. 2020. Early childhood caries status and its associated factors among young children in a rural area of Cambodia." Pediatric Dental Journal 30(1): 17-23.
45. Kubota Y, San Pech N, Durward C, Ogawa H. 2020. Association between Early Childhood Caries and Maternal Factors among 18- to 36-month-old Children in a Rural Area of Cambodia. Oral health & preventive dentistry 18(1): 973-980.
46. Lara J S, Romano A, Murisi PU, Tedesco T K, Mendes F M, Soto-Rojas A E, Alonso C, Campus G. Impact of early childhood caries severity on oral health-related quality of life among preschool children in Mexico: A cross-sectional study. Int J Paediatr Dent. 2022 May;32(3):334-343. doi: 10.1111/ipd.12889. Epub 2021 Dec 16. PMID: 34358390.
47. Leelataweewud P, Jirarattanasopha V, Ungchusak C and Vejvithee W. 2021. Psychometric evaluation of the Thai version of the Early Childhood Oral Health Impact Scale (Th-ECOHIS): a cross sectional validation study. BMC Oral Health 21(1): 64.
48. Li J, Fan W, Zhou Y, Wu L, Liu W, Huang S. 2020. The status and associated factors of early childhood caries among 3- to 5-year-old children in Guangdong, Southern China: a provincial cross-sectional survey. BMC Oral Health 20(1): 265.
49. Li Y, Wulaerhan J, Liu Y, Abudureyimu A, Zhao J. 2017. Prevalence of severe early childhood caries and associated socioeconomic and behavioral factors in Xinjiang, China: a cross-sectional study. BMC Oral Health 17(1): 144.
50. Meyer F, Karch A, Schlinkmann K M, Dreesman J, Horn J, Rübsamen N, et al. 2017. Sociodemographic determinants of spatial disparities in early childhood caries: An ecological analysis in Braunschweig, Germany. Community Dentistry and Oral Epidemiology 45(5): 442-448.
51. Moimaz S A, Borges H C, Saliba O, Garbin C A, Saliba N A et al. 2016. Early Childhood Caries: Epidemiology, Severity and Sociobehavioural Determinants. Oral health & preventive dentistry 14(1): 77-83.
52. Munoz L V. Dissertacion. Asociación de la caries dental con el nivel de conocimiento sobre salud oral de los padres/madres de niños de 3 a 7 años de edad en una institucion educativa publica del distrito de Arte-Vtare. 2014. https://repositorioacademico.upc.edu.pe/handle/10757/322243?show=full
53. Musinguzi N, Kemoli A, Okullo I. Prevalence and Treatment Needs for Early Childhood Caries Among 3-5-Year-Old Children From a Rural Community in Uganda. Front Public Health. 2019 Sep 18;7:259. doi: 10.3389/fpubh.2019.00259. PMID: 31620416; PMCID: PMC6759934.
54. Nagarajappa R, Satyarup D, Naik D, Dalai R P et al. 2020. Feeding practices and early childhood caries among preschool children of Bhubaneswar, India. European Archives of Paediatric Dentistry 21(1): 67
55. Naidu R, Nunn J, Donnelly-Swift et al. 2016. Oral health-related quality of life and early childhood caries among preschool children in Trinidad. BMC Oral Health 16(1)
56. Naidu R, Nunn J, Kelly A et al. 2013. Socio-behavioural factors and early childhood caries: a cross-sectional study of preschool children in central Trinidad. BMC Oral Health 13:30.
57. Nakayama Y, Mori M, 2015. Association of environmental tobacco smoke and snacking habits with the risk of early childhood caries among 3-year-old Japanese children. Journal of Public Health Dentistry 75(2): 157-162.
58. Nakayama Y, Mori M, 2015. Association between nocturnal breastfeeding and snacking habits and the risk of early childhood caries in 18- to 23-month-old Japanese children. Journal of epidemiology / Japan Epidemiological Association 25(2): 142-147.
59. Nakayama Y, Ohnishi H, Mori M. 2019. Association of Environmental Tobacco Smoke with the Risk of Severe Early Childhood Caries among 3-Year-Old Japanese Children. Caries Research 53(3): 268-274.
60. Ndekero T S, Carneiro L C, Masumo R M et al. 2021. Prevalence of early childhood caries, risk factors and nutritional status among 3-5-yearold preschool children in Kisarawe, Tanzania. PLOS ONE 16.
61. Nguyen Y H T, Ueno M, Zaitsu T, Nguyen T, Kawaguchi Y.et al. 2018. Early childhood caries and risk factors in Vietnam. Journal of Clinical Pediatric Dentistry 42(3): 173-181.
62. Nobile C G, Fortunato L, Bianco A, Pileggi C, Pavia M et al. 2014. Pattern and severity of early childhood caries in Southern Italy: a preschool-based cross-sectional study. BMC Public Health 14: 206.
63. Olatosi O O, Li M, Alade A A, Oyapero A, Busch T, Pape J, et al. 2021. Replication of GWAS significant loci in a sub-Saharan African Cohort with early childhood caries: a pilot study. BMC Oral Health 21(1):274
64. Olczak-Kowalczyk D, Gozdowski D, Kaczmarek U et al. 2020. Factors associated with early childhood caries in Polish three-year-old children. Oral Health and Preventive Dentistry 18(4): 833-842.
65. Özen B, Van Strijp A J, Özer L, Olmus H, Genc A, Cehreli S B et al. 2016. Evaluation of Possible Associated Factors for Early Childhood Caries and Severe Early Childhood Caries: A Multicenter Cross-Sectional Survey. The Journal of clinical pediatric dentistry 40(2): 118-123.
66. Ozler C O, Tekcicek M U, Ozdemir P, Dogan B G et al. 2018. Pufa index and related factors among 36- to 71-month-old children in Turkey: A cross-sectional study." Oral Health and Preventive Dentistry 16(5): 467-472.
67. Parisotto T M, Steiner-Oliveira C, de Souza-e-Silva C M, Peres R C R, Rodrigues L K A, Nobre-dos-Santos M et al. 2012. Assessment of cavitated and active non-cavitated caries lesions in 3- to 4-year-old preschool children: A field study. International Journal of Paediatric Dentistry 22(2): 92-99.
68. Pattanaporn K, Saraithong P, Khongkhunthian S, Aleksejuniene J, Laohapensang P, Chhun N et al. 2013. Mode of delivery, mutans streptococci colonization, and early childhood caries in three- to five-year-old Thai children. Community Dentistry and Oral Epidemiology 41(3): 212-223.
69. Peltzer K, Mongkolchati A. 2015. Severe early childhood caries and social determinants in three-year-old children from Northern Thailand: a birth cohort study. BMC Oral Health 15: 108.
70. Pereira J L, Caramelo F, Soares A D, Cunha B, Gil A M, Costa A L. 2021. Prevalence and sociobehavioural determinants of early childhood caries among 5-year-old Portuguese children: a longitudinal study. European archives of paediatric dentistry: official journal of the European Academy of Paediatric Dentistry 22(3): 399-408.
71. Perera P J, Fernando M P, Warnakulasooriya T D, Ranathunga N. 2014. Effect of feeding practices on dental caries among preschool children: A hospital based analytical cross- sectional study. Asia Pacific Journal of Clinical Nutrition 23(2): 272-277.
72. Pikramenou V, Dimitraki D, Zoumpoulakis M, Verykouki E, Kotsanos N. 2016. Association between dental caries and body mass in preschool children. European Archives of Paediatric Dentistry 17(3): 171-175.
73. Pinto G D S, Azevedo M S, Goettems M L, Correa M B, Pinheiro R T, Demarco F F. 2017. Are Maternal Factors Predictors for Early Childhood Caries? Results from a Cohort in Southern Brazil. Brazilian Dental Journal 28(3): 391-397.
74. Prakasha Shrutha S, Vinit G B G, Giri K Y, Alam S et al. 2013. Feeding practices and early childhood caries: A cross-sectional study of preschool children in Kanpur District, India. ISRN Dentistry 2013.
75. Qadri G, A. Nourallah and C. H. Splieth et al. 2012. Early childhood caries and feeding practices in kindergarten children. Quintessence international (Berlin, Germany: 1985) 43(6): 503-510.
76. Rajab L D, Abdullah R B. 2020. Impact of dental caries on the quality of life of preschool children and families in Amman, Jordan. Oral Health and Preventive Dentistry 18(3): 571-582.
77. Rashid N B. 2019. "Maternal dental health knowledge and its relation to the dental caries experience of their children in mamyzawa camp of refugees in Erbil, Iraq." Acta Medica Academica 48(3): 294-302 %U http://www.ama.ba/index.php/ama/article/view/387/pdf.
78. Šačić L, Marković N, Arslanagić Muratbegović A, Zukanović A, Kobašlija S. The prevalence and severity of early childhood caries in preschool children in the Federation of Bosnia and Herzegovina. Acta Med Acad. 2016 May;45(1):19-25. doi: 10.5644/ama2006-124.152. PMID: 27284794.
79. Sankeshwari R M, Ankola A V, Tangade P S, Hebbalet M. 2013. Association of socio-economic status and dietary habits with early childhood caries among 3- to 5-year-old children of Belgaum city. European archives of paediatric dentistry: official journal of the European Academy of Paediatric Dentistry 14(3): 147-153.
80. Schulz B, Wolter I, Schiffner U. 2022. Kariesprävalenz und Karieserfahrung bei 3- bis 6-järigen Kindern in Hamburg. Oralprophylaxe Kinderzahnheilkd 2022 44: 34-39.
81. Seminario A L, Jumani K, Velan E, Scott J M, Latimer J, Schroth R J. 2018. Suboptimal Serum Vitamin D Associated with Early Childhood Caries in Special Health Care Needs Children. Journal of dentistry for children (Chicago, Ill.) 85(3): 93-101.
82. Severino M, Caruso S, Ferrazzano G F, Pisaneschi A, Fiasca F, Caruso S et al. 2021. Prevalence of Early Childhood Caries (ECC) in a paediatric italian population: An epidemiological study. European Journal of Paediatric Dentistry 22(3): 189-198.
83. Shakavets N. 2018. Preventive Programmes of Early Childhood Caries in Belarus. International reviews: clinical practice and health 3. p. 26-29.
84. Sharma K, Gupta K, Gaur A, Sharma A, Pathania V, Thakur V. 2019. A cross-sectional study to assess the prevalence of early childhood caries and associated risk factors in preschool children in district Mandi, Himachal Pradesh. Journal of Indian Society of Pedodontics and Preventive Dentistry 37(4): 339-344.
85. Sitthisettapong T, Tasanarong P, Phantumvanit P. Strategic Management of Early Childhood Caries in Thailand: A Critical Overview. Front Public Health. 2021 Jun 11; 9:664541. doi: 10.3389/fpubh.2021.664541. PMID: 34178924; PMCID: PMC8226079.
86. Songa M A S, Saliba N A, Saliba T A, Chiba F Y, Moimaz S A S. Analysis of the Dental Caries Epidemiological Profile in Children of Benguela city, Angola. Oral Health Prev Dent. 2022 Mar 14;20(1):141-148. doi: 10.3290/j.ohpd.b2805501. PMID: 35285603.
87. Sun L. 2020. The association between postpartum depression and early childhood caries. Acta Odontologica Scandinavica 78(5): 352-357.
88. Tsang C, Sokal-Gutierrez K, Patel P, Lewis B, Huang D, Ronsin K, Baral A, Bhatta A, Khadka N, Barkan H, Gurung S. Early Childhood Oral Health and Nutrition in Urban and Rural Nepal. Int J Environ Res Public Health. 2019 Jul 10;16(14):2456. doi: 10.3390/ijerph16142456. PMID: 31295932; PMCID: PMC6678585.
89. Tubert-Jeannin S, Leger S, Manevy R. 2012. Addressing children's oral health inequalities: caries experience before and after the implementation of an oral health promotion program. Acta Odontologica Scandinavica 70(3): 255-264.
90. Turton B, Chher T, Sabbah W, Durward C, Hak S, Lailou A. 2019. Epidemiological survey of early childhood caries in Cambodia. BMC Oral Health 19(1): 107.
91. Turton B, Durward C, Manton D, Bach K, Yos C. 2016. Socio-behavioral risk factors for early childhood caries (ECC) in Cambodian preschool children: a pilot study. European Archives of Paediatric Dentistry 17(2): 97-105.
92. Tušek I, O'Rourke B, Lekić C, Tušek J, Tušek B. 2020. Early childhood caries in multilingual community. Central European journal of public health 28(4): 286-291.
93. Wang X, Wie Z, Li Q, Mei L. 2017. A longitudinal study of early childhood caries incidence in Wenzhou preschool children. BMC Oral Health 17(1): 105.
94. Warren J J, Blanchette D, Dawson D V, Marshall T A, Phipps K R, Starr D, et al. 2016. Factors associated with dental caries in a group of American Indian children at age 36 months. Community Dentistry and Oral Epidemiology 44(2): 154-161.
95. Wong H M, McGrath C P J, King N M, Lo E M C. 2011. Oral health-related quality of life in Hong Kong preschool children. Caries Research 45(4): 370-376.
96. Wu R Y, Cao G Z, Feng V, Feng X P, Chen X, Han X L. 2020. Risk factors of dental caries among young children in Pudong New District, Shanghai. Shanghai kou qiang yi xue = Shanghai journal of stomatology 29(4): 405-409.
97. Wulaerhan J, Abudureyimu A, Bao X L, Zhao J, 2014. Risk determinants associated with early childhood caries in Uygur children: a preschool-based cross-sectional study. BMC Oral Health 14: 136.
98. Zambrano O, Fong L, Rivera L, Calatayud E, Hernández J, Maldonado A, Rojas-Sánchez F, Principe S, Pérez G, Navarro T, Acevedo A. Impacto de la caries de infancia temprana en la calidad de vida del niño zuliano y su familia. Odous Científica. 2015; 16(2): 8-17
99. Zhang M, Zhang X, Zhang Y, Li Y, Shao C, Xiong S et al. 2020. Assessment of risk factors for early childhood caries at different ages in Shandong, China and reflections on oral health education: a cross-sectional study. BMC Oral Health 20(1): 139.
100. Zhu H, Ying X, Zhu F, Huang C, Yu Y. 2020. Early childhood caries and related risk factors: A cross-sectional study of children in Zhejiang Province, China. International Journal of Dental Hygiene 18(4): 352-361.
